# Growth hormone receptor-deficient pigs resemble the pathophysiology of human Laron syndrome and reveal altered activation of signaling cascades in the liver

**DOI:** 10.1016/j.molmet.2018.03.006

**Published:** 2018-03-15

**Authors:** Arne Hinrichs, Barbara Kessler, Mayuko Kurome, Andreas Blutke, Elisabeth Kemter, Maren Bernau, Armin M. Scholz, Birgit Rathkolb, Simone Renner, Sebastian Bultmann, Heinrich Leonhardt, Martin Hrabĕ de Angelis, Hiroshi Nagashima, Andreas Hoeflich, Werner F. Blum, Martin Bidlingmaier, Rüdiger Wanke, Maik Dahlhoff, Eckhard Wolf

**Affiliations:** 1 Chair for Molecular Animal Breeding and Biotechnology, Gene Center and Department of Veterinary Sciences, LMU Munich, Feodor-Lynen-Str. 25, 81377 Munich, Germany; 2 Center for Innovative Medical Models (CiMM), Department of Veterinary Sciences, LMU Munich, Hackerstr. 27, 85764 Oberschleißheim, Germany; 3 Meiji University International Institute for Bio-Resource Research, 1-1-1 Higashimita, Tama, Kawasaki, 214-8571, Japan; 4 Institute of Veterinary Pathology, Center for Clinical Veterinary Medicine, LMU Munich, Veterinärstr. 13, 80539 Munich, Germany; 5 Livestock Center of the Veterinary Faculty, LMU Munich, St.-Hubertus-Str. 12, 85764 Oberschleißheim, Germany; 6 German Center for Diabetes Research (DZD), Helmholtz Zentrum München, Ingolstädter Landstr. 1, 85764 Neuherberg, Germany; 7 Human Biology and Bioimaging, Faculty of Biology, Biocenter, LMU Munich, Großhaderner Str. 2, 82152 Planegg-Martinsried, Germany; 8 Institute of Experimental Genetics, Helmholtz Zentrum München, and Chair of Experimental Genetics, Technical University of Munich, Ingolstädter Landstr. 1, 85764 Neuherberg, Germany; 9 Cell Signaling Unit, Institute of Genome Biology, Leibniz Institute for Farm Animal Biology (FBN), Wilhelm-Stahl-Allee 2, 18196 Dummerstorf, Germany; 10 University Children`s Hospital, University of Giessen, Feulgenstr.12, 35392 Gießen, Germany; 11 Endocrine Laboratory, Medizinische Klinik und Poliklinik IV, Klinikum der Universität München, Ziemssenstr. 1, 80336 Munich, Germany; 12 Laboratory for Functional Genome Analysis (LAFUGA), Gene Center, LMU Munich, Feodor-Lynen-Str. 25, 81377 Munich, Germany

**Keywords:** Growth hormone receptor, Laron syndrome, Pig model, Dwarfism, Hypoglycemia, Insulin-like growth factor 1, Signaling, 4EBP1, eukaryotic initiation factor 4E binding protein 1, aa, amino acid, AKT, serine-threonine protein kinase, AMPK, AMP-activated protein kinase, CRISPR/Cas, clustered regularly interspaced short palindromic repeats/CRISPR-associated, DAB, 3,3′-diaminobenzidine, DXA, dual-energy X-ray absorptiometry, eIF4E, eukaryotic translation initiation factor 4E, ELISA, enzyme-linked immunosorbent assay, GH, growth hormone, GHR, growth hormone receptor, GSK3B, glycogen synthase 3 beta, HDL, high-density lipoprotein, HOMA, homeostatic model assessment, HSL, hormone-sensitive lipase, IGF1, insulin-like growth factor 1, IGFBP, IGF-binding protein, IgG, immunoglobulin G, INSR, insulin receptor, IRS1, insulin receptor substrate 1, JAK2, Janus kinase 2, LS, Laron syndrome, LSM, least squares mean, LDL, low-density lipoprotein, LEPR, leptin receptor, LPL, lipoprotein lipase, MAPK, mitogen-activated protein kinase, MRI, magnetic resonance imaging, mTOR, mechanistic target of rapamycin, mTORC, mTOR complex, PCR, polymerase chain reaction, PI3K, phosphoinositide 3 kinase, PPARG, peroxisome proliferator-activated receptor gamma, RIA, radioimmunoassay, S6K, protein S6 kinase 1, SE, standard error, sgRNA, single guide RNA, STAT, signal transducer and activator of transcription, TBS, Tris-buffered saline

## Abstract

**Objective:**

Laron syndrome (LS) is a rare, autosomal recessive disorder in humans caused by loss-of-function mutations of the growth hormone receptor (*GHR*) gene. To establish a large animal model for LS, pigs with *GHR* knockout (KO) mutations were generated and characterized.

**Methods:**

CRISPR/Cas9 technology was applied to mutate exon 3 of the *GHR* gene in porcine zygotes. Two heterozygous founder sows with a 1-bp or 7-bp insertion in *GHR* exon 3 were obtained, and their heterozygous F1 offspring were intercrossed to produce *GHR*-KO, heterozygous *GHR* mutant, and wild-type pigs. Since the latter two groups were not significantly different in any parameter investigated, they were pooled as the GHR expressing control group. The characterization program included body and organ growth, body composition, endocrine and clinical-chemical parameters, as well as signaling studies in liver tissue.

**Results:**

*GHR*-KO pigs lacked GHR and had markedly reduced serum insulin-like growth factor 1 (IGF1) levels and reduced IGF-binding protein 3 (IGFBP3) activity but increased IGFBP2 levels. Serum GH concentrations were significantly elevated compared with control pigs. *GHR*-KO pigs had a normal birth weight. Growth retardation became significant at the age of five weeks. At the age of six months, the body weight of *GHR*-KO pigs was reduced by 60% compared with controls. Most organ weights of *GHR*-KO pigs were reduced proportionally to body weight. However, the weights of liver, kidneys, and heart were disproportionately reduced, while the relative brain weight was almost doubled. *GHR*-KO pigs had a markedly increased percentage of total body fat relative to body weight and displayed transient juvenile hypoglycemia along with decreased serum triglyceride and cholesterol levels. Analysis of insulin receptor related signaling in the liver of adult fasted pigs revealed increased phosphorylation of IRS1 and PI3K. In agreement with the loss of GHR, phosphorylation of STAT5 was significantly reduced. In contrast, phosphorylation of JAK2 was significantly increased, possibly due to the increased serum leptin levels and increased hepatic leptin receptor expression and activation in *GHR*-KO pigs. In addition, increased mTOR phosphorylation was observed in *GHR*-KO liver samples, and phosphorylation studies of downstream substrates suggested the activation of mainly mTOR complex 2.

**Conclusion:**

*GHR*-KO pigs resemble the pathophysiology of LS and are an interesting model for mechanistic studies and treatment trials.

## Highlights

• GHR-deficient pigs reveal postnatal growth retardation, disproportionate organ growth and an increased total body fat content.
• GHR-deficient pigs show markedly reduced serum IGF1 and IGFBP3 levels, and transient juvenile hypoglycemia.
• Increased expression and phosphorylation of IRS1 in liver of adult GHR-deficient pigs suggest increased insulin sensitivity.
• Increased phosphorylation of JAK2 in liver of GHR-deficient pigs may be explained by higher serum leptin levels and activation of hepatic LEPR.

## 1 Introduction

Laron syndrome (LS) is a rare, autosomal recessive, hereditary disorder caused by loss-of-function mutations in the growth hormone receptor (*GHR*) gene (https://www.omim.org/entry/600946), initially described as a syndrome of primary growth hormone (GH) resistance or insensitivity ([1]; reviewed in [2], [3]). As a consequence, LS patients have low levels of insulin-like growth factor 1 (IGF1) and – due to the lack of feedback inhibition of GH secretion – high levels of GH [3]. A few hundred cases of LS have been reported world-wide, caused by a variety of *GHR* mutations (reviewed in [4]). Among them is an isolated, more homogeneous population of GHR deficient patients in Ecuador with only two distinct mutations of the *GHR* gene [5], [6], [7].

The main clinical feature is short stature. In addition, LS patients may exhibit reduced muscle strength and endurance, hypoglycemia in infancy, delayed puberty, obesity, and distinct facial features, including a protruding forehead, sunken bridge of the nose, and blue sclerae (reviewed in [3], [8]). The standard treatment of LS is long-term application of recombinant IGF1, which increases growth velocity and improves adult height, but it may lead to a spectrum of side effects, in particular hypoglycemia ([9], [10]; reviewed in [11]).

A particularly interesting observation in LS patients is their reduced incidence of malignancies ([12], [13]; reviewed in [8]). In addition, LS patients from the cohort in Ecuador have been shown to be protected against the development of type 2 diabetes despite severe obesity [7].

Although mechanistic studies have been performed in cell lines derived from LS patients and healthy controls [7], [14], animal models are of pivotal importance for understanding the pathophysiology of LS *in vivo*. In particular, GHR-deficient mice [15] have provided new insights into the consequences of GH insensitivity for body and organ growth, body composition, endocrine and metabolic functions, and reproduction, as well as aging and life expectancy (reviewed in [16]). More recently, inducible/tissue-specific *Ghr* knockout (KO) mouse models have helped to define the specific roles of GHR in liver, muscle, and adipose tissue and revealed interesting differences compared with constitutive *Ghr* KO mice [17], [18]. However, due to their small size, short life expectancy and physiological differences compared with humans, findings from mouse models may be difficult to extrapolate to the clinical situation of LS patients. In general, genetically tailored pig models are useful to bridge the gap between proof-of-concept studies in rodent models and clinical studies in patients (reviewed in [19], [20]). Thus, we have developed a GHR-deficient (*GHR*-KO) pig model and show that it resembles important aspects of LS pathophysiology and reveals altered activation of signaling cascades in the liver.

## 2 Materials and methods

### 2.1 Generation of *GHR* mutant pigs using CRISPR/Cas

All animal procedures in this study were approved by the responsible animal welfare authority (Regierung von Oberbayern; permission 55.2-1-54-2532-70-12) and performed according to the German Animal Welfare Act and Directive 2010/63/EU on the protection of animals used for scientific purposes.

For CRISPR/Cas-assisted *GHR* gene disruption using a single guide RNA (sgRNA) specific for exon 3 sequence 5′-TTCATGCCACTGGACAGATG-3′, a corresponding oligonucleotide was cloned into the pEX-A-U6-gRNA vector as described previously [21]. Cas9 mRNA and sgRNA were *in vitro*-transcribed using the Ambion Maxiscript SP6 kit (Thermo Fisher Scientific).

Porcine zygotes (German landrace background) were produced *in vitro* as described previously [22], and Cas9 mRNA (50 ng/μL) and sgRNA (100 ng/μL) was injected into their cytoplasm 8.5–9.5 h after *in vitro* fertilization. Recipient gilts were synchronized in the estrous cycle by oral administration of 4 mL Altrenogest (Regumate^®^; MSD Animal Health) for 15 days, followed by intramuscular injection of 750 IU ECG (Intergonan^®^; MSD Animal Health) and 750 IU HCG (Ovogest^®^; MSD Animal Health) after an additional 24 and 104 h, respectively. Embryo transfer was performed laparoscopically into one oviduct [23], [24]. Pregnancy was confirmed by ultrasonographic examination first on day 21 and again 4–6 weeks later.

Genomic DNA was isolated from tail tips of piglets using the Wizard DNA Extraction Kit (Promega). *GHR* mutations were detected by sequencing a *GHR* exon 3 PCR product obtained using primers GHR_Fw 5′-acc gct ctg aag ctg tga cc-3′ and GHR_Rv 5′-cac cct cag ata ctc tca tgc-3′. Based on the detected mutations, an *Xcm*I restriction fragment length polymorphism assay was established, yielding fragments of 203 bp and 441 bp for wild-type *GHR* and a single fragment of 644 bp for the mutated *GHR* sequence.

Two female founder animals with different frameshift mutations were mated with wild-type boars to generate heterozygous F1 offspring. Heterozygous offspring of the same founder were intercrossed to obtain homozygous animals (*GHR*-KO) with the respective *GHR* mutations. The resulting pedigrees are shown in Suppl. Fig. 1.

### 2.2 Ligand immunostaining of porcine GHR

Liver and kidney tissue of *GHR*-KO and control pigs was fixed overnight in 4% formaldehyde and routinely embedded in paraffin. Paraffin sections were dewaxed, and endogenous peroxidase and biotin were blocked with 1% H_2_O_2_ in Tris-buffered saline (TBS) for 15 min and by using the avidin/biotin blocking kit (no. SP-2001; Vector Laboratories), respectively. After blocking with 0.5% fish gelatin for 30 min, the slides were incubated in 0.5 μg/mL recombinant rat GH (no. 16343667, ImmunoTools) in 0.2% fish gelatin solution overnight at 4 °C. They were then washed 3 times for 5 min in TBS and incubated in goat anti-rat GH polyclonal antibody solution (dilution 1:2,400, no. AF1566, R&D Systems) for 6 h at room temperature. After 3 washing steps in TBS (10 min each), the slides were incubated in biotinylated rabbit anti-goat IgG solution (dilution 1:100, no. BA-5000, Vector Laboratories) for 1 h at room temperature, washed 3 times for 10 min in TBS, and finally incubated with horseradish peroxidase-labelled avidin biotin complex for 30 min (no. PK-6100, VECTASTAIN Elite ABC-Peroxidase kit, Vector Laboratories). Immunoreactivity was visualized using 3,3′-diaminobenzidine tetrahydrochloride dihydrate (DAB) (brown color). Nuclear counterstaining was performed with Mayer's hemalum (blue color). As specificity controls for the ligand immunohistochemistry assay, rat GH as well as rat GH plus primary antibody were omitted.

### 2.3 Blood collection

Animals were fasted overnight (16 h) before blood collection from the jugular vein. After clotting for 30 min at room temperature, serum was separated by centrifugation (1200 × *g*) for 20 min at 6 °C and stored at −80 °C until analysis. For repeated blood sampling required to analyze GH secretion profiles, central venous catheters (Argon Careflow™; Merit Medical) were surgically inserted through the external ear vein. Blood samples were collected every 30 min for 9 h, starting at 11 a.m., and processed for serum collection as described above. During the test, the animals were fed regularly and had free access to water.

### 2.4 Clinical chemistry, hormone assays, IGFBP ligand blot analysis

Clinical-chemical parameters in serum were determined using a Cobas 311 system (Hitachi) or an AU480 autoanalyzer (Beckman-Coulter) and adapted reagents from Roche Diagnostics or Beckman-Coulter, respectively. Serum GH concentrations were measured by an ELISA for rat/mouse GH (EZRMGH-45K; Merck) that cross-reacts with porcine GH. To calculate the area under the GH curve, values below the quantification limit of the assay (<0.07 ng/mL) were arbitrarily set to 0.07 ng/mL. IGF1 levels in serum were determined by RIA after dissociation of IGF1 from IGFBPs by acidification and blocking the IGF1 binding sites with an excess of IGF2 [25]. IGFBP ligand blot analysis of serum samples was performed as described previously [26] using serial dilutions of recombinant human IGFBP3 (41/38 kDa), IGFBP2 (32 kDa), IGFBP5 (29 kDa) and IGFBP4 (24 kDa) for quantification. Plasma insulin was determined using a species-specific RIA (Merck Millipore) as previously described [27]. Blood glucose levels were determined immediately using a Precision Xceed^®^ glucometer and Precision XtraPlus^®^ test strips (Abbott) [28]. Serum leptin levels were measured using a multi-species leptin radioimmunoassay (Cat. # XL-85K; EMD Millipore Corporation) that has been validated for porcine samples [29].

### 2.5 Growth parameters and body composition

Body weight and body length (distance between tip of the snout and tail root in straightened animals) of *GHR*-KO and control pigs were determined at weekly intervals. Relative body length was calculated by dividing the body length by the cube root of the body weight to retain the same dimensions. Since not all animals could be weighed/measured at exactly the same ages, raw data were adjusted by linear interpolation to defined ages/time points.

The percentage of total body fat was determined in 6-month-old *GHR*-KO and control pigs using dual-energy X-ray absorptiometry (DXA; Lunar *i*DXA, GE Healthcare) as previously described [30]. In addition, magnetic resonance imaging (MRI; Magnetom Open, Siemens) [31] was performed to visualize and determine the muscle to fat ratio as the area of longissimus dorsi muscle divided by the area of its overlying back fat at the last rib.

### 2.6 Necropsy

*GHR*-KO and control pigs were euthanized at 6 months of age under anesthesia by intravenous injection of T61^®^ (Intervet) and immediately subjected to necropsy. Organs were dissected and weighed to the nearest mg. Tissue samples were collected as described previously [32] and routinely fixed in neutral buffered formalin solution (4%) for 24 h or frozen immediately on dry ice and stored at −80 °C for molecular profiling. Formalin-fixed tissue specimens were embedded in paraffin. Muscle sections were stained with hematoxylin and eosin (H&E).

### 2.7 Immunoblot analysis of signaling cascades

The concentrations and phosphorylation status of GHR-related signaling molecules in the liver were evaluated by Western blot analyses as described previously [33]. Briefly, liver tissue samples were homogenized in Laemmli extraction buffer, and the protein content was determined by the bicinchoninic acid protein assay. Forty micrograms of total protein was separated by SDS-PAGE and transferred to PVDF membranes (Millipore) by electro-blotting. Membranes were washed in TBS with 0.1% Tween-20 and blocked in 5% w/v fat-free milk powder (Roth) for 1 h. The membranes were then washed again and incubated in 5% w/v BSA (Roth) solution with the appropriate primary antibodies overnight at 4 °C. The antibodies and concentrations used are listed in Suppl. Table 1. After washing, the membranes were incubated in 5% w/v fat-free milk powder solution with the secondary antibody (donkey anti-rabbit; 1:2000; GE Healthcare) for 1 h. Bound antibodies were detected using the ECL Advance Western Blotting Detection Kit (GE Healthcare) and appropriate films from the same supplier. Band intensities were quantified using the ImageQuant software package (GE Healthcare).

### 2.8 Statistical analyses

Longitudinal data for body weight and body length or relative body length respectively were analyzed using PROC MIXED (SAS 8.2), taking the effects of pig line (#2529; #2533), group (*GHR*-KO; control), sex, age, and interaction group*age into account. Least squares means (LSMs) and standards errors (SEs) of LSMs were calculated for group*age and compared using Student's t-test. Data for glucose homeostasis and serum lipid concentrations were analyzed using PROC GLM (SAS 8.2), taking the effects of group, sex, age and the interaction group*age into account. LSMs and SEs were calculated for group*age and compared using Student's t-test. Body composition, organ weight and clinical-chemical data were analyzed using PROC GLM taking the effects of group and sex into account. LSMs and SEs were calculated for groups and compared using Student's t-test. IGFBP ligand blot and Western immunoblot data were evaluated for significant differences between *GHR*-KO and control pigs using the Mann-Whitney U test.

## 3 Results

### 3.1 Generation of a growth hormone receptor-deficient pig model

**Figure 1:**
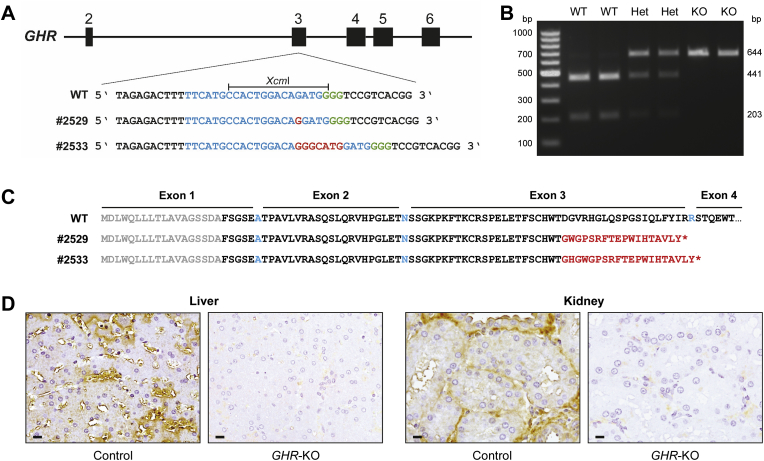
Generation of a GHR-deficient pig model using CRISPR/Cas technology. (A) Partial DNA sequence of *GHR* exon 3. The sgRNA binding site is indicated in blue and the protospacer adjacent motif (PAM) in green. Insertions (red) of 1 bp (founder #2529) or 7 bp (founder #2533) lead to a shift of the reading frame. WT = wild type. (B) Restriction fragment length polymorphism analysis to detect the WT *GHR* sequence as well as monoallelic (Het) and biallelic (KO) mutations. (C) Partial amino acid sequences encoded by the WT and mutant *GHR* alleles. The signal peptide is shown in gray, WT GHR aa sequence in black (aa encoded by adjacent non-symmetrical exons in blue), missense aa sequence in red, and the premature termination codon as an asterisk. (D) Ligand immunohistochemistry demonstrating the absence of functional GHR (brown staining in control) in *GHR*-KO pigs. Chromogen: DAB; counterstain: Mayer's hemalum; bar = 10 μm.

We employed CRISPR/Cas9 technology to generate *GHR* knockout (*GHR-*KO) pigs as a large animal model for Laron syndrome (LS). *In vitro*-transcribed RNA encoding Cas9 and sgRNA specific for *GHR* exon 3 was injected into *in vitro* fertilized porcine oocytes, which were transferred to recipient gilts. In total, 8 piglets were born, of which 3 showed monoallelic mutations in the *GHR* gene. Two female founder animals carried monoallelic insertions of 1 bp (#2529) or 7 bp (#2533) (Figure 1A). The two founder animals were mated with wild-type boars to establish pedigrees for phenotypic analyses of GHR-deficient (*GHR-*KO) vs. GHR-expressing F2 animals (Suppl. Fig. 1). The two lines were kept separate except in one experiment to test the fertility of *GHR-*KO pigs. Wild-type, heterozygous, and *GHR-*KO littermates were identified by PCR and restriction fragment length polymorphisms of the mutated and wild-type *GHR* alleles (Figure 1B). Since we did not observe significant differences between heterozygous *GHR* mutant and wild-type animals (Suppl. Fig. 2), they were pooled and used as the GHR-expressing control group.

### 3.2 Homozygous frameshift mutations in *GHR* exon 3 result in GHR deficiency

The insertion of 1 bp (#2529) or 7 bp (#2533) leads to a shift in the reading frame in *GHR* exon 3. The mutant *GHR* transcripts encode the 18-aa signal peptide and 51 aa of the extracellular GHR domain, followed by an 18-aa or a 20-aa missense sequence and premature termination codon after 87 aa (#2529) or 89 aa (#2533) (Figure 1C).

The presence of GHR was investigated in liver and kidney sections since these tissues naturally express high levels of GHR [34]. To evaluate GH binding, the sections were incubated with recombinant GH, and bound GH was detected using specific antibodies. This ligand immunohistochemistry approach showed strong GH binding in control tissues, while GH binding was absent in tissue sections from *GHR-*KO animals (Figure 1D).

### 3.3 Decreased serum IGF1 and IGFBP3, and increased IGFBP2 in *GHR*-KO pigs

**Figure 2:**
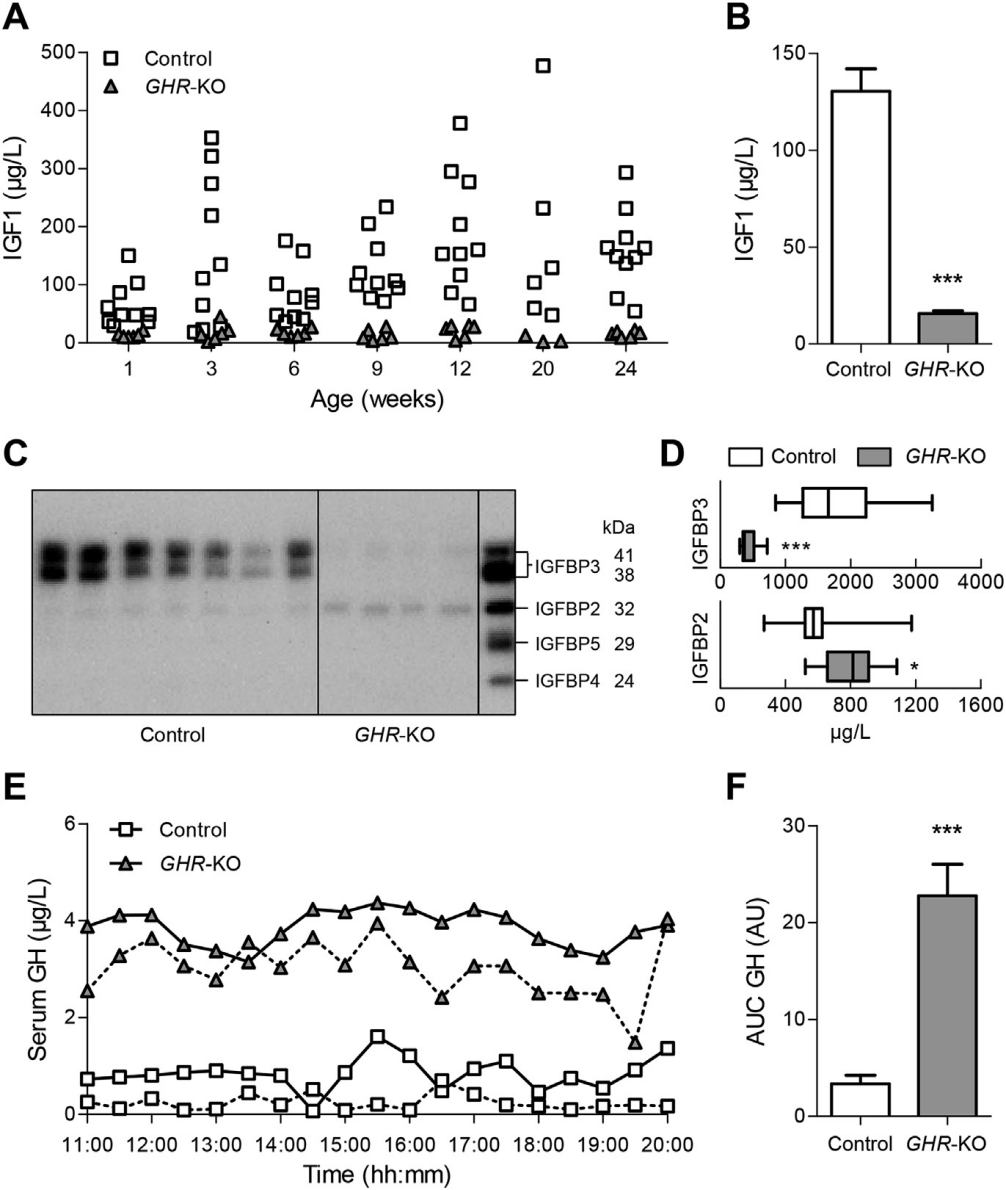
Serum IGF1, IGFBP and GH concentrations of *GHR*-KO compared with control pigs. (A) Scatter plot of serum IGF1 levels of *GHR*-KO and control pigs over time. (B) Means and standard deviations of all serum IGF1 values displayed in panel A (*GHR*-KO: n = 42; control: n = 69). (C) Representative IGFBP ligand blot. Right lane displays recombinant human IGFBP3 (41/38 kDa), IGFBP2 (32 kDa), IGFBP5 (29 kDa) and IGFBP4 (24 kDa). (D) Quantification of IGFBP3 and IGFBP2 in serum from *GHR*-KO (n = 10) and control pigs (n = 12). The figure shows medians, 25th and 75th percentiles (box), and extremes (whiskers). (E) Representative GH secretion profiles of two female *GHR*-KO and two female control pigs. (F) Area under the GH curve (AUC; means and standard deviations for 6 female *GHR*-KO and 5 female/1 male control pigs). AU = arbitrary units. *p < 0.05; ***p < 0.001.

*GHR-*KO pigs of all ages showed a marked reduction in serum insulin-like growth factor 1 (Figure 2A,B). IGF binding proteins (IGFBPs) were evaluated by ligand blot analysis in 6-month-old animals using a dilution series of recombinant human IGFBPs for quantification (Figure 2C). IGFBP3 was significantly decreased in *GHR-*KO pigs (426 ± 41 μg/L vs. 1775 ± 205 μg/L in control animals; p < 0.0001), while IGFBP2 was significantly increased (799 ± 53 μg/L vs. 607 ± 66 μg/L in control animals; p = 0.0272) (Figure 2D).

### 3.4 *GHR*-KO pigs have high levels of circulating GH

To evaluate effects of GHR deficiency on the pulsatile secretion of GH, we collected serial blood samples at 30-min intervals over a period of 9 h (starting at 11 a.m.) from five female and one male 9-month-old *GHR-*KO pigs and from six age-matched female controls. Serum GH levels of *GHR-*KO pigs were high with partially preserved pulsatility (area under the GH curve was increased 6.8-fold in *GHR-*KO compared with control pigs), indicating a disturbance in the negative feedback control of GH secretion (Figure 2E,F; Suppl. Fig. 3).

### 3.5 *GHR*-KO pigs show severe growth retardation

**Figure 3:**
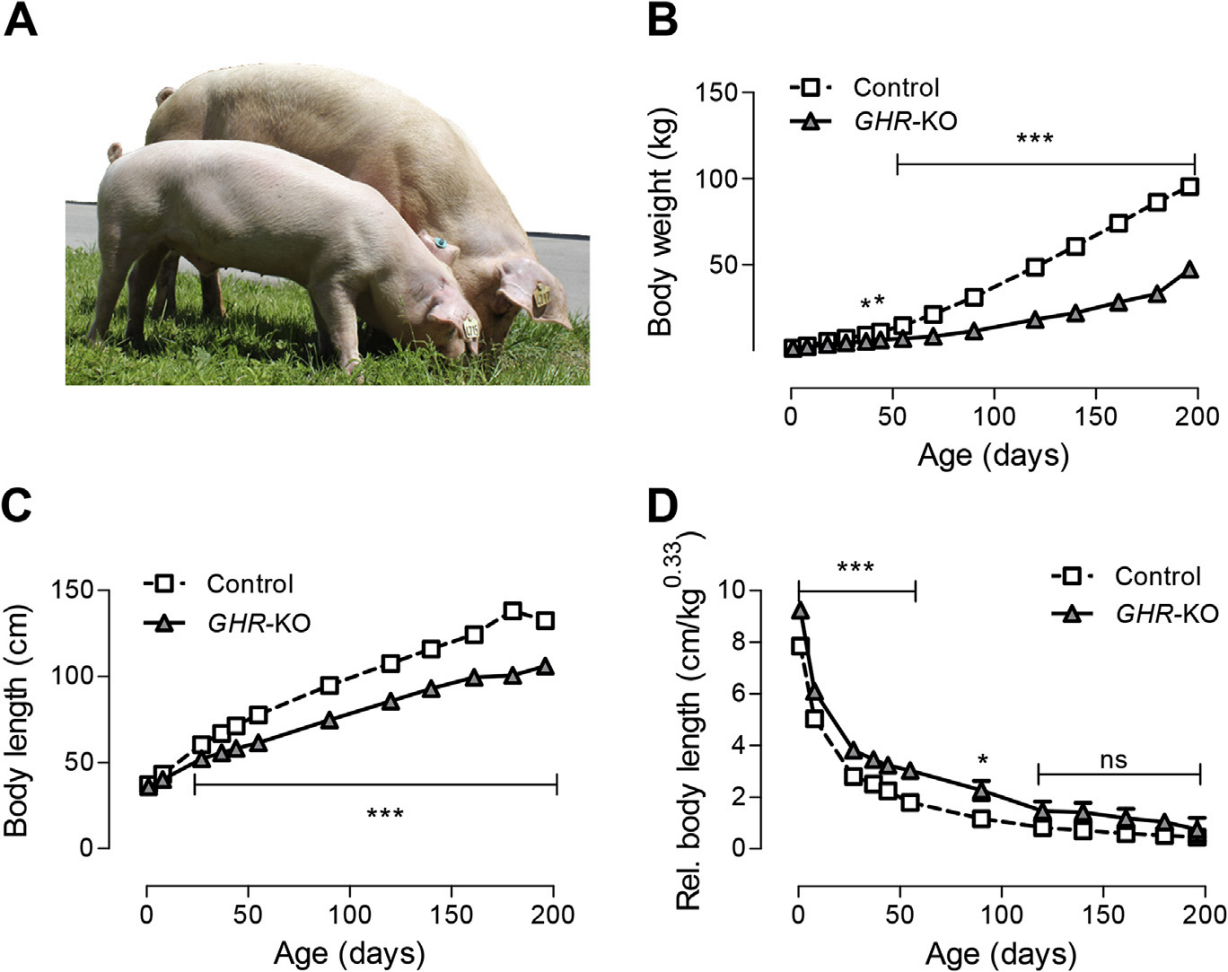
Body weight gain and growth of *GHR*-KO compared with control pigs. (A) *GHR*-KO pig (front) and control littermate aged 6 months. (B) Body weight gain. (C) Body length. (D) Relative body length (body length divided by the cube root of body weight). These parameters were determined in 12 *GHR*-KO and 25 control pigs. Panels A–D show least squares means (LSMs) and standard errors of LSMs estimated for group*age (see 2.8 for the statistical model). *p < 0.05; **p < 0.01; ***p < 0.001; ns = not significant.

The birth weight of *GHR-*KO piglets did not differ from control littermates. First significant growth retardation became obvious at five weeks of age (p = 0.045), leading to a 62% reduction in body weight of 6-month-old *GHR-*KO pigs (33.0 ± 1.5 kg) compared with age-matched control animals (86.2 ± 1.1 kg; p < 0.0001) (Figure 3A,B). Body length at birth did not differ between *GHR-*KO and control piglets (33.9 ± 1.4 cm vs. 37.3 ± 1 cm; p = 0.4068). Significant differences in body length appeared at four weeks of age (52.2 ± 1.4 cm in *GHR-*KO vs. 60.3 ± 1 cm in control animals; p < 0.0001). At six months of age, the body length of *GHR-*KO pigs was reduced by 27% compared with control pigs (100.6 ± 1.6 cm vs. 138.1 ± 1.4 cm; p < 0.0001) (Figure 3C). Up to an age of four months, weight gain was more affected than linear growth by GHR deficiency, as indicated by an increased relative body length (body length divided by the cube root of body weight) of *GHR-*KO pigs (Figure 3D). No growth parameters exhibited significant sex-related differences.

### 3.6 *GHR*-KO pigs show an increased proportion of body fat and a reduced ratio of muscle to fat tissue

**Figure 4:**
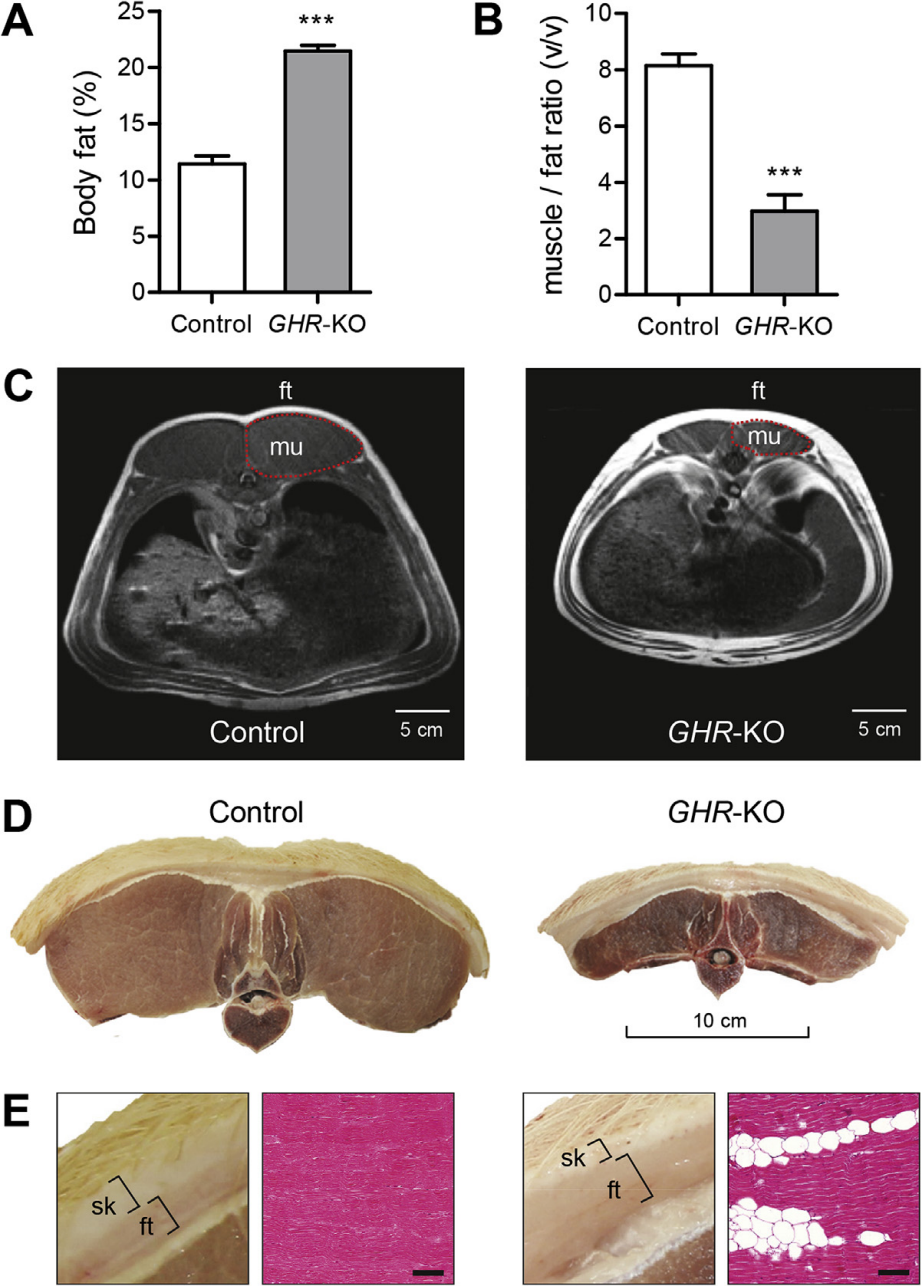
Body composition of 6-month-old *GHR*-KO compared with control pigs. (A) DXA analysis revealed a significantly higher amount of total body fat in *GHR*-KO pigs. (B) The calculated ratio of muscle to fat tissue from MRI images at the last rib revealed a significant shift towards fat tissue in *GHR*-KO pigs (*GHR*-KO: n = 12; control: n = 25; ***p < 0.001). Panels A and B show least squares means (LSMs) and standard errors of LSMs estimated for the 2 groups (see 2.8 for the statistical model). (C) Representative magnetic resonance images used to evaluate the volume of the longissimus dorsi muscle (mu) and its overlying back fat (ft) at the last rib in *GHR*-KO and control pigs. Note the larger subcutaneous and visceral fat depots in *GHR*-KO pigs. (D) Representative macroscopic cross-sections of the first lumbar vertebra, the two longissimus dorsi muscles and the overlying back fat and skin. (E) Higher magnification of D showing an increased ratio of subcutaneous fat (ft) to skin (sk) thickness in a *GHR*-KO compared with a control pig. Histological section (H&E stain) showing an increased amount of intramuscular fat in *GHR*-KO pigs (bar = 100 μm).

DXA analysis revealed a markedly increased percentage of total body fat in 6-month-old *GHR-*KO pigs (21.5 ± 0.7% vs. 11.4 ± 0.5% in age-matched control animals; p < 0.0001) (Figure 4A). While female pigs in the control group displayed significantly higher body fat content than male pigs (13.2 ± 0.7% vs. 9.6 ± 0.7%; p = 0.0009), no sex-related differences were observed in *GHR-*KO pigs (22.9 ± 1% in males vs. 22 ± 1% in females; p = 0.4530). To determine the ratio of muscle to fat tissue, MRI scans were performed at the location of the last rib, and the volume ratio of the longissimus dorsi muscle and its overlying back fat was calculated (Figure 4B,C). *GHR-*KO pigs showed a significantly reduced muscle to fat tissue ratio compared with control pigs (3.0 ± 0.6 vs. 8.2 ± 0.4; p < 0.0001) (Figure 4B). No sex-related differences were observed in this parameter (Suppl. Table 2). The MRI findings were confirmed upon necropsy, showing a marked increase in the thickness of the subcutaneous fat tissue and a reduction in the size of the longissimus dorsi muscle in *GHR*-KO pigs (Figure 4D,E). Histological sections of skeletal muscle samples from *GHR*-KO pigs revealed markedly increased numbers of adipocyte section profiles between muscle fibers (Figure 4E).

### 3.7 Disproportionate organ growth of *GHR*-KO pigs

**Figure 5:**
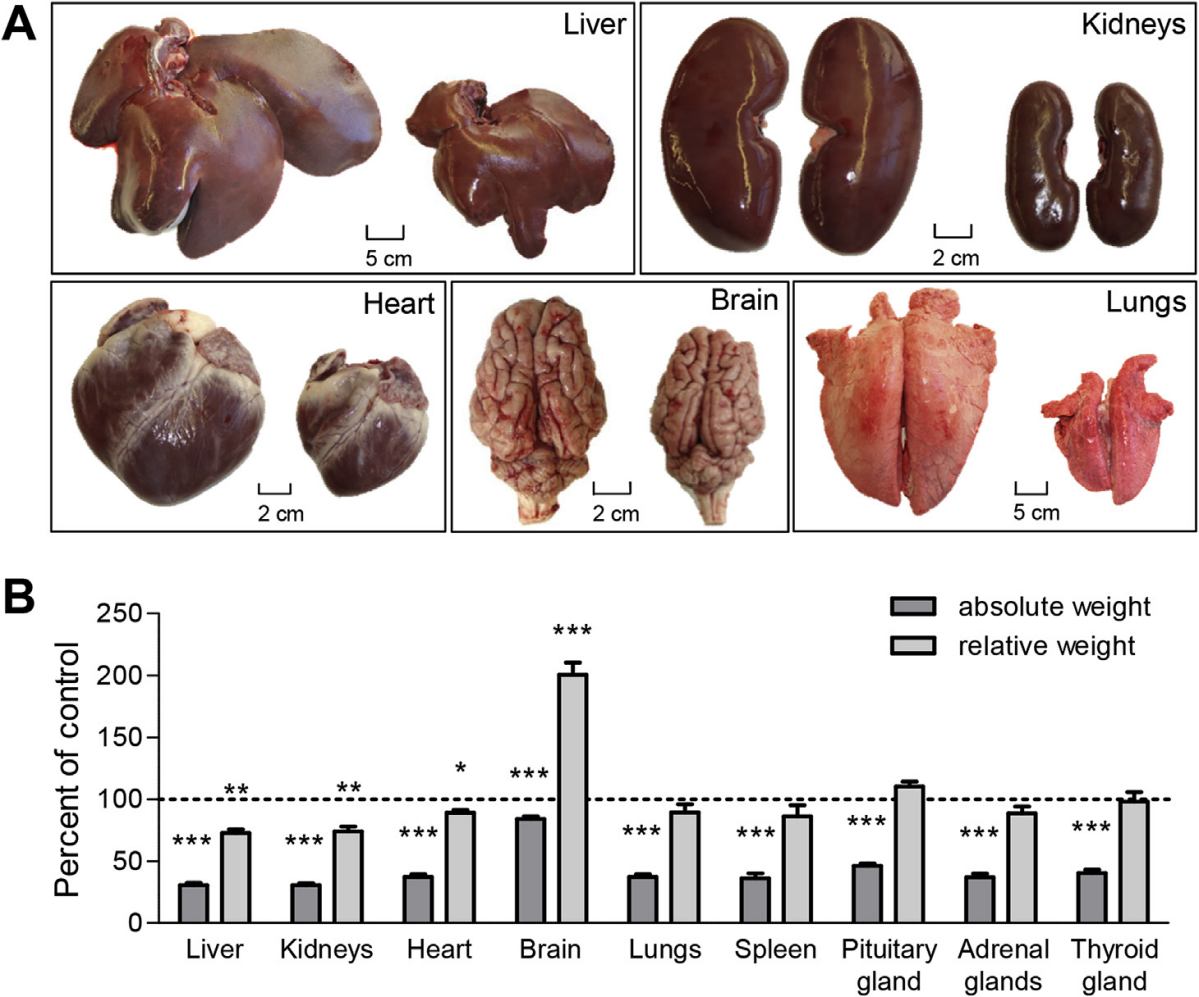
Disproportionate organ growth in *GHR*-KO compared with control pigs. GHR-deficiency led to a proportionate and disproportionate reduction in organ sizes. (A) Representative organs from control (left) and *GHR*-KO pigs (right). (B) Relative differences between *GHR*-KO and control pigs in absolute organ weights and in organ weight-to-body weight ratios (relative organ weights). These parameters were determined in 9 *GHR*-KO and 25 control pigs, and least squares means (LSMs) and standard errors of LSMs were estimated for the 2 groups (see 2.8 for the statistical model). *p < 0.05; **p < 0.01; ***p < 0.001.

In 6-month-old *GHR-*KO pigs, absolute weights of all organs were significantly smaller than in age-matched control animals (Figure 5, Suppl. Table 3). Most organ weights of *GHR-*KO pigs were reduced proportionally to body weight. However, the relative weights of liver (73% of control animal relative liver weight; p = 0.0005), kidneys (73% of control animal relative kidney weight; p = 0.0002) and heart (87% of control animal relative heart weight; p = 0.0119) were significantly reduced, while relative brain weight was doubled (200% of control animal relative brain weight; p < 0.0001) (Figure 5, Suppl. Table 3).

### 3.8 Male and female *GHR*-KO pigs are fertile

The ovaries of 6-month-old *GHR*-KO gilts did not show obvious morphological differences from control ovaries. Mating of an 8-month-old *GHR*-KO boar (line #2529) with a *GHR*-KO sow (line #2533) of the same age resulted in a litter of 6 healthy *GHR*-KO piglets (Suppl. Fig. 4A). Their birth weight tended to be reduced in comparison to *GHR*-KO piglets derived from heterozygote × heterozygote matings (on average 0.75 kg compared with 1.3 kg). However, the animals showed catch-up growth and achieved a higher body weight at 6 months of age than *GHR*-KO offspring from heterozygous *GHR*-KO parents (43.8 ± 1.3 kg vs. 33.0 ± 2.2 kg; p < 0.0001; Suppl. Fig. 4B).

### 3.9 *GHR*-KO pigs show transient hypoglycemia, while insulin levels remain unaffected

**Figure 6:**
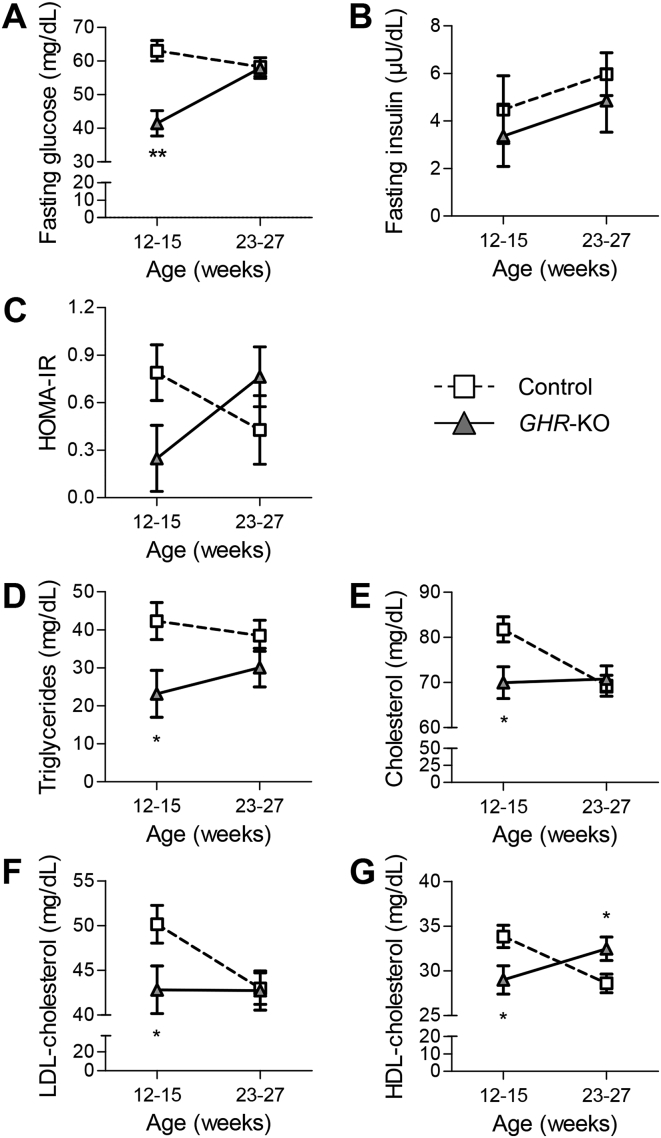
Age-dependent changes in glucose and lipid homeostasis parameters in *GHR*-KO and control pigs. (A) Transient juvenile hypoglycemia in *GHR*-KO pigs. (B) Unchanged serum insulin concentrations. (C) Initially lower, then higher HOMA-IR score (interaction group*age: p < 0.05). Serum concentrations of (D) triglycerides, (E) cholesterol, (F) low-density lipoprotein (LDL)-cholesterol, and (G) high-density lipoprotein (HDL)-cholesterol levels were significantly lower in young *GHR*-KO pigs than in age-matched controls, but normalized with age. HDL-cholesterol levels of 23- to 27-week-old *GHR*-KO pigs were even higher than in their control littermates. At least 6 animals per group and age-class were investigated. Panels A–G show least squares means (LSMs) and standard errors of LSMs estimated for group*age (see 2.8 for the statistical model). *p < 0.05; **p < 0.01.

Young *GHR-*KO pigs (12–15 weeks old) showed significantly reduced fasting blood glucose levels (41.5 ± 3.8 mg/dL vs. 63.1 ± 3.1 mg/dL in age-matched control animals; p = 0.0001), while this difference disappeared in older animals (23–27 weeks) (Figure 6A). Fasting serum insulin levels of *GHR-*KO pigs did not differ from control pigs at any age (Figure 6B). In addition, the homeostatic model assessment (HOMA) for evaluating insulin resistance was calculated from fasting glucose and insulin concentrations. In agreement with their low fasting blood glucose concentrations, HOMA-IR values of young *GHR-*KO pigs tended to be reduced (0.25 ± 0.20 vs. 0.76 ± 0.19 in age-matched control pigs; p = 0.0635), but increased to 0.76 ± 0.18 in the older age group (p = 0.0839), when controls had a HOMA-IR of 0.43 ± 0.20 (p = 0.2584; Figure 6C). Analysis of variance revealed a significant (p < 0.05) interaction of group*age.

### 3.10 Serum lipid levels are reduced in young *GHR*-KO pigs

Young *GHR-*KO pigs (aged 12–15 weeks) revealed significantly decreased serum concentrations of triglycerides (23.2 ± 6.8 mg/dL vs. 42.3 ± 4.9 mg/dL in controls; p = 0.021; Figure 6D), cholesterol (70 ± 3.5 mg/dL vs. 81.8 ± 2.8 mg/dL in controls; p = 0.0129; Figure 6E), low-density lipoprotein (LDL)-cholesterol (42.8 ± 2.7 mg/dL vs. 50.1 ± 2.1 mg/dL in controls; p = 0.0392; Figure 6F) and high-density lipoprotein (HDL)-cholesterol (29.0 ± 1.6 mg/dL vs. 33.8 ± 1.3 mg/dL in controls; p = 0.0224; Figure 6G). In older *GHR-*KO pigs (23–27 weeks), serum triglyceride, cholesterol and LDL-cholesterol concentrations were similar to those of control pigs, while serum HDL-cholesterol levels were increased (32.5 ± 1.3 mg/dL vs. 28.6 ± 1.0 mg/dL in controls; p = 0.0272) (Figure 6D–G). Excluding triglycerides, all investigated lipid parameters were significantly (p < 0.0002) affected by sex, with higher levels in female than in male animals (Suppl. Table 4).

### 3.11 Additional alterations of clinical-chemical parameters in *GHR*-KO pigs

To screen for changes in organ functions and metabolic pathways, a broad spectrum of clinical-chemical parameters in serum of 6-month-old *GHR-*KO and control pigs were analyzed. While all parameters remained within physiological ranges, *GHR-*KO pigs displayed lower levels of creatinine (91.2 ± 6.5 μmol/L vs. 126.4 ± 5.0 μmol/L in controls; p = 0.0004) but higher levels of urea (7.4 ± 0.5 mmol/L vs. 4.3 ± 0.3 mmol/L in controls; p < 0.0001) (Suppl. Table 5).

### 3.12 *GHR*-KO pigs display significant changes in the activation of hepatic signaling cascades

Since the liver is a major target tissue of insulin and GH, we evaluated changes in associated signaling cascades by Western blot analyses of liver samples from fasted 6-month-old *GHR*-KO and control animals.

**Figure 7:**
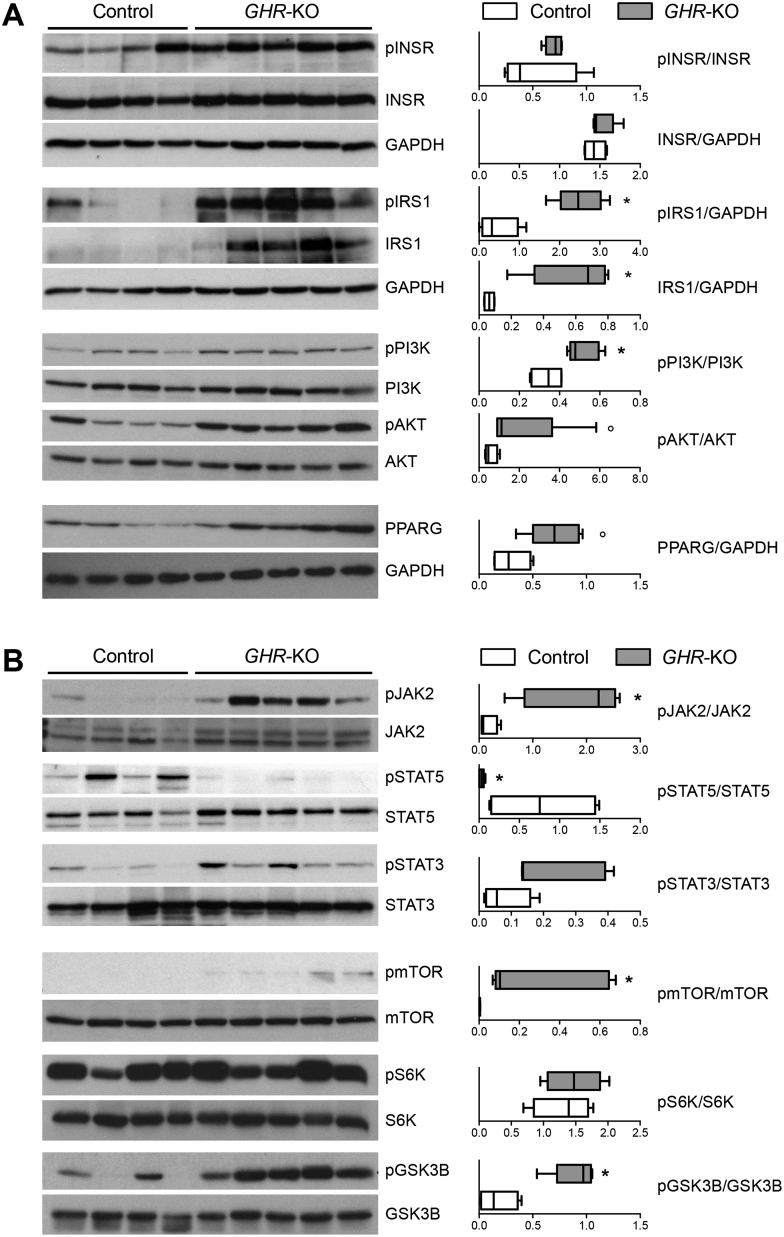
Western blot analysis of signaling cascades in liver samples of 6-month-old fasted *GHR*-KO (n = 5) and control pigs (n = 4). (A) Insulin receptor-related signaling pathway and PPARG. (B) GHR- and mTOR-related signaling pathways. The box plots show medians, 25th and 75th percentiles (box), and extremes (whiskers). *p < 0.05; °p = 0.0635; evaluated using the Mann–Whitney U test.

The total amounts of insulin receptor (INSR) and of phosphorylated INSR were unchanged in *GHR*-KO liver samples. In contrast, the concentrations of total and phosphorylated insulin receptor substrate 1 (IRS1) were significantly (p = 0.0159) increased in liver samples from *GHR*-KO vs. control pigs. The investigation of signal transducers downstream of the INSR revealed significantly increased phosphorylation of phosphoinositide 3 kinase (PI3K) and a trend (p = 0.0635) toward an increase in serine/threonine protein kinase AKT phosphorylation in *GHR*-KO liver samples. Furthermore, a trend (p = 0.0635) toward increased levels of total peroxisome proliferator-activated receptor gamma (PPARG) was observed (Figure 7A).

*GHR*-KO liver samples showed significantly increased phosphorylation levels of Janus kinase 2 (JAK2). The phosphorylation levels of signal transducer and activator of transcription 5 (STAT5) were significantly reduced, while STAT3 phosphorylation showed a tendency to increase (p = 0.1111). Phosphorylation of STAT1 was not significantly different between *GHR-*KO and control pigs, whereas significantly increased phosphorylation levels of mitogen-activated protein kinase (MAPK) were detected (Suppl. Fig. 5).

In addition, liver extracts from *GHR*-KO pigs showed a significant increase in phosphorylated mechanistic target of rapamycin (mTOR), which was not detected in control liver samples. To distinguish between the activation of mTOR complex 1 (mTORC1) and 2 (mTORC2), we analyzed several specific up- and downstream signal transducers for each complex [35], [36] (Figure 7B; Suppl. Fig. 5).

A key element of mTORC1 action is protein S6 kinase 1 (S6K), which phosphorylates S6. *GHR-*KO liver samples did not show increased phosphorylation of S6K (Figure 7B). Phosphorylation of other effectors downstream of mTORC1 — eukaryotic initiation factor 4E binding protein 1 (4EBP1) and eukaryotic translation initiation factor 4E (eIF4E) — was unchanged in *GHR*-KO liver samples (Suppl. Fig. 5). Phosphorylation of AMP-activated protein kinase (AMPK), an inhibitor of mTORC1, was significantly increased in *GHR-*KO liver tissue (Suppl. Fig. 5). Collectively these data suggest that mTORC1 is not activated in liver of *GHR*-KO pigs.

**Figure 8:**
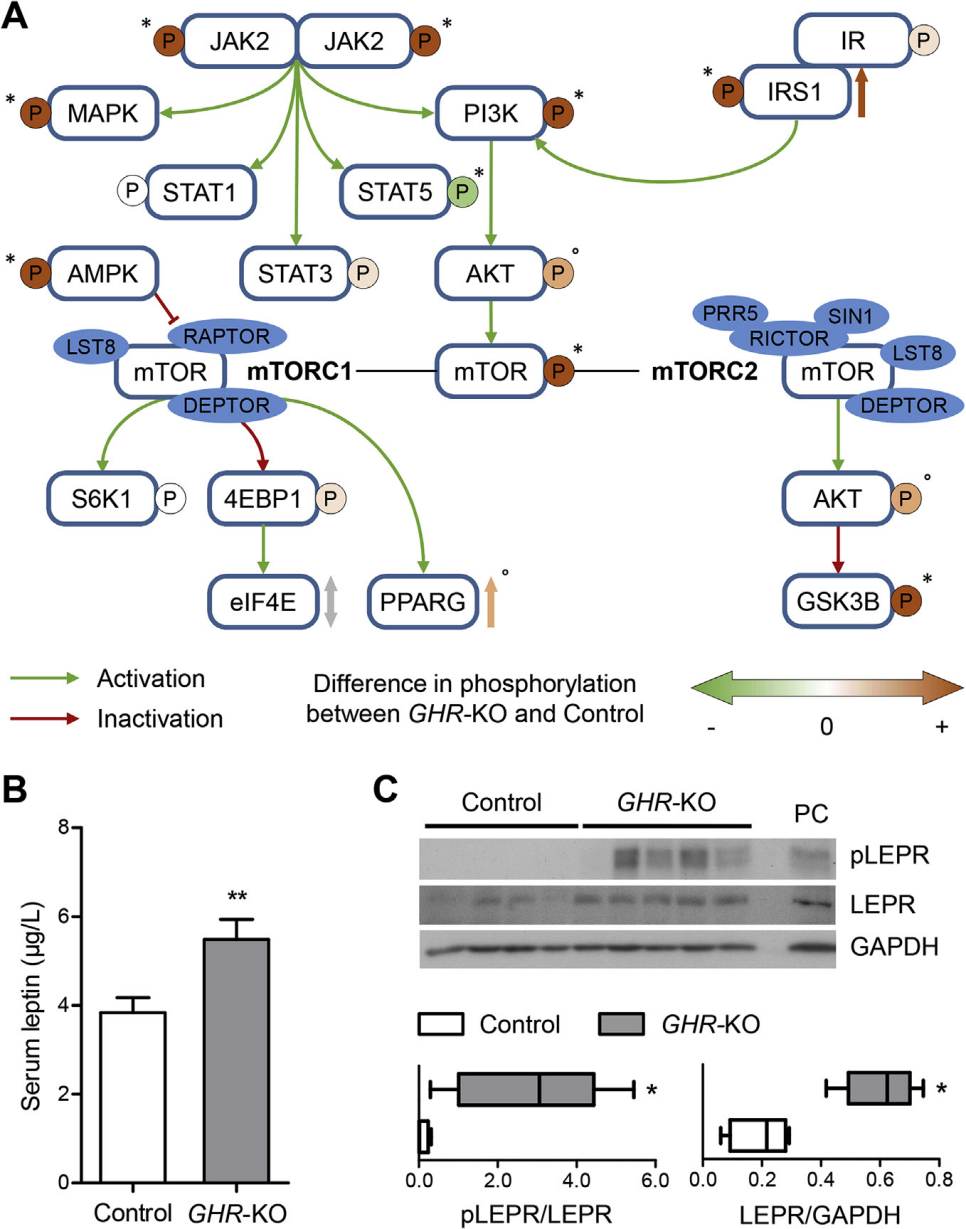
(A) Schematic summary of the changes in phosphorylation in INSR- and GHR-related signaling molecules in liver samples of *GHR*-KO compared with control pigs. *p < 0.05; °p = 0.0635; evaluated using the Mann–Whitney U test. (B) Significantly increased fasting serum leptin concentrations in 6-month-old *GHR*-KO vs. control pigs. The figure shows the estimated least squares means (LSMs) and standard errors of the LSMs for the two groups, taking into account the effect of sex (9 male/13 female control pigs; 6 male/6 female *GHR*-KO pigs). **p < 0.01 for the effect of group (PROC GLM). (C) Significantly increased expression and phosphorylation of LEPR in liver samples from *GHR*-KO compared with control pigs. PC = protein lysate from choroid plexus of a wild-type pig used as positive control. *p < 0.05; evaluated using the Mann–Whitney U test.

In contrast, one of the most important downstream substrates of mTORC2 — glycogen synthase 3 beta (GSK3B) – showed significantly increased phosphorylation in *GHR-*KO liver tissue (Figure 7B). Furthermore, the phosphorylation levels of potent regulators of mTORC2 action, PI3K and AKT, were also increased (Figure 7A). These findings suggest that mTORC2 is activated in the liver of GHR-deficient pigs. A summary of these findings is provided in Figure 8A.

### 3.13 Serum leptin levels and hepatic leptin receptor activation are increased in *GHR*-KO pigs

Considering the expected reduced STAT5 phosphorylation but unexpected increased JAK2 phosphorylation in the liver of *GHR*-KO pigs, we measured serum leptin levels and hepatic leptin receptor (LEPR) activation. LEPR signaling involves activation of JAK2 and STAT3, but not STAT5 (reviewed in [37]). Overall, serum leptin concentrations of 6-month-old fasted *GHR*-KO pigs were significantly elevated compared with the controls (Figure 8B), with higher levels in females than in males (Suppl. Table 2). In addition, LEPR phosphorylation was significantly increased in *GHR*-KO vs. control liver samples (Figure 8C).

## 4 Discussion

This study reports a new large animal model for GHR deficiency (Laron syndrome, LS). Previously described pig models with impaired GH function, which are either GHR-deficient [38] or express a dominant negative GHR [39], were established on a minipig background that is already growth impaired and, thus, may not resemble all pathophysiological consequences of human LS. Therefore, we established our GHR-deficient model on a German landrace background with the physiological growth potential of domestic pigs and performed a comprehensive characterization of body and organ growth, body composition, endocrine and metabolic changes, and signaling cascades in liver.

Our GHR-deficient pig model exhibits important hallmarks of human LS, such as insensitivity to GH, low circulating IGF1 concentrations, and reduced postnatal body and organ growth. Similar findings have been reported for minipig models with impaired GH function [38], [39]. Our study provides a more comprehensive phenotypic analysis, including serial GH measurements, effects on serum IGFBPs, age-dependent changes in glucose homeostasis and lipid profiles, and analyses of hepatic signaling cascades.

Circulating IGF1 originates in large part from the liver, as shown by liver specific *Igf1* KO mice [40], and is complexed by high-affinity IGFBPs, with IGFBP3 and an acid labile subunit (ALS) forming a 150-kDa complex, which is the main reservoir of IGF1 in the bloodstream (reviewed in [41], [42]). ALS is directly regulated by GH, and reduced ALS levels are observed in GHR-deficient patients [43]. The production of IGFBP3 is also stimulated by GH [44], and GHR deficiency in mice and humans leads to reduced levels of IGFBP3 in the circulation [15], [45]. In agreement with these reports, serum IGFBP3 concentrations of *GHR*-KO pigs were significantly reduced, presumably leading to a decrease in the IGF1 reservoir in the circulation and thus to a shortened half-life. In addition, the concentration of IGFBP2 was increased in serum of *GHR*-KO pigs. Studies in transgenic IGFBP2-overexpressing mice have shown that IGFBP2 inhibits the growth of normal [46] and even of GH-overexpressing mice [47]. Therefore, the reduced growth of *GHR*-KO pigs most likely results from a combination of a decline in the production and half-life of circulating IGF1 and, possibly, IGF1 sequestration by inhibitory IGFBP2.

Circulating IGF1 is also responsible for the feedback inhibition of pituitary GH secretion (reviewed in [48]). In accordance with this concept, *GHR*-KO pigs with low serum IGF1 levels showed high serum GH concentrations with partially preserved pulsatility. Serial blood samples for the analysis of hormone profiles can be obtained easily from pigs equipped with permanent central venous catheters [49], whereas serial blood sampling in mice is more difficult.

Interestingly, a growth deficiency phenotype of *GHR*-KO pigs was observed no earlier than postnatal week 5, which indicated that GH was not required as promoter of intrauterine and early postnatal growth. This finding is consistent with observations in infants with LS [50] and in neonatal *Ghr* KO mice [15]. Instead, intrauterine growth depends on IGF2 and IGF1, with the latter acting independently of GH during this period of development [51]. An essential role of GH as a postnatal growth promoter is established during the maturation of the endocrine growth axis, which is associated with an increase in expression of hepatic GHR [52]. In general, intrauterine maturation of endocrine functions in pigs is similar to humans, whereas rodents are born in a more immature state (reviewed in [53]).

In agreement with observations in human LS patients [54] and *Ghr* KO mice [55], *GHR*-KO pigs displayed an increase in total body fat and a decrease in the muscle to fat ratio. Adipose tissue mass is determined by the storage and removal of triglycerides in adipocytes. A recent study in which human adipocyte lipid age was determined by measuring ^14^C from nuclear bomb tests revealed that triglycerides are renewed up to 6 times during the average adipocyte life-span of 10 years [56]. In *GHR*-KO pigs, physiological adipocyte lipid turnover is apparently disturbed at different levels. First, the lipolytic action of GH via an increase in adipose tissue hormone-sensitive lipase (HSL) activity (reviewed in [16], [57]) is lost in the absence of GHR. Second, the synthesis of storage lipids in adipocytes is likely to increase in *GHR*-KO pigs. This phenomenon requires hydrolysis by lipoprotein lipase (LPL) of the triglyceride component of circulating chylomicrons and very low-density lipoproteins (VLDL) into free fatty acids and 2-monoacylglycerol, which can be taken up by adipocytes. Adipose tissue LPL activity is increased by insulin but inhibited by GH and sex steroids (reviewed in [58]). Reduced serum triglyceride levels in young *GHR*-KO pigs (12–15 weeks) with normal insulin levels, but lacking the counteracting effects of GH and sex steroids, may reflect an increased use of triglycerides for lipid synthesis in adipocytes, leading to an increase in total body fat. In older *GHR*-KO pigs (23–27 weeks), the increased total body fat content may lead to decreased triglyceride turnover of adipocytes [56], limiting the use of circulating triglycerides for storage lipid synthesis in adipocytes. Furthermore, the animals became sexually mature, with sex steroids potentially inhibiting adipose tissue LPL and the hydrolysis of serum triglycerides, thus resulting in normal serum triglyceride levels. Decreased serum cholesterol levels, as observed in young *GHR*-KO pigs, have also been reported in human LS patients [3] and *Ghr* KO mice (reviewed in [16]).

Measurements of organ weights in *GHR*-KO pigs revealed that growth of liver, kidneys, and heart is particularly dependent on GHR/GH action, as their relative weights were significantly decreased in comparison to control pigs. Reduced relative liver and kidney weights have also been observed in *Ghr* KO mice [55], and interestingly LS patients have disproportionately reduced cardiac dimensions [59]. The important role of the GH/IGF1 system in the growth of these organs is supported by their disproportionate overgrowth in conditions of GH/IGF1 excess, as in GH-overexpressing transgenic mice [60], [61] and in patients with acromegaly [62]. In contrast, brain growth is less dependent on intact GHR signaling, as shown by a relatively moderate reduction in absolute brain weight and a marked increase in the relative brain weight of *GHR*-KO pigs. Based on similar observations in *Ghr* KO mice, Sjogren et al. [63] speculated that a large proportion of brain growth occurs relatively early in development in a GH-independent stage.

Patients with Laron syndrome exhibit delayed puberty, but they are able to achieve full sexual development and reproduce [2]. In *Ghr* KO mice, delayed puberty and reduced litter sizes have been observed. The latter has been attributed to reduced ovarian function due to the lack of IGF1 (reviewed in [16]). To assess the fertility of *GHR*-KO pigs, a *GHR-*KO boar and a *GHR-*KO sow were mated, resulting in a litter of 6 healthy piglets. Although not significant, the mean birth weight of these piglets was 40% lower than that of *GHR-*KO piglets from heterozygote x heterozygote mating, most likely because of the limited fetal growth capacity in a smaller mother. Interestingly, *GHR-*KO piglets from homozygous parents showed catch-up growth and had a 33% higher body weight at six months than *GHR-*KO piglets from heterozygous parents. An explanation for this increased growth performance may be hybrid vigor, potentially resulting from the mating of *GHR*-KO pigs from two different lines (Suppl. Fig. 1).

*GHR*-KO pigs showed juvenile hypoglycemia that normalized when the animals reached sexual maturity. Hypoglycemia is a hallmark of juvenile age LS [3], [64] and has been mainly explained by the lack of stimulatory GH effects on hepatic gluconeogenesis (reviewed in [57]). Contrasting observations have been obtained concerning the role of altered insulin sensitivity in the development of juvenile hypoglycemia (reviewed in [65]). While no evidence for increased insulin sensitivity, and even some cases of insulin-resistant diabetes mellitus, have been reported in the Israeli cohort of LS patients [64], [66], individuals from the Ecuadorian LS cohort are more insulin-sensitive than their *GHR* intact relatives [67], and no cases of diabetes mellitus were reported [7]. The reasons for this discrepancy remain elusive [65]. Although HOMA-IR is not a routinely established parameter to assess insulin (in)sensitivity in pigs (reviewed in [68]), the trend (p = 0.0635) toward lower HOMA-IR scores in young *GHR*-KO vs. control pigs suggests that increased insulin sensitivity is a contributing factor to juvenile hypoglycemia, as also observed in some *Ghr* KO mouse models ([69]; reviewed in [16]). While the HOMA-IR scores of *GHR*-KO pigs increased with age, and tended to be higher in sexually mature *GHR*-KO than in age-matched control pigs (interaction group*age: p < 0.05), there was no evidence for insulin resistance. Interestingly, we observed increased levels of total and phosphorylated IRS1 in liver samples from fasted adult *GHR*-KO pigs, which could represent a mechanism for increased insulin sensitivity. Similar observations and conclusions were obtained in GH-deficient Ames dwarf mice with increased insulin sensitivity [70]. Future studies involving state-of-the-art *in vivo* measurements of gluconeogenesis [71] and investigation of insulin sensitivity using hyperinsulinemic, euglycemic clamp studies in juvenile and sexually mature *GHR*-KO and control pigs will help to clarify the relative contributions of these mechanisms to juvenile hypoglycemia in LS and its normalization in adult LS patients. Such studies can be performed more accurately in pigs than in rodent models due to their larger size [49].

While all clinical-chemical parameters measured in the serum of *GHR*-KO pigs remained within the normal reference ranges for pigs, creatinine levels were reduced and serum urea concentrations were increased compared with the control pigs. The serum creatinine concentration correlates with muscle mass [72], which explains the reduced levels in *GHR*-KO pigs and in LS patients [3]. Increased serum concentrations of urea, the end product of amino acid catabolism in mammals, have also been detected in *Ghr* KO mice [69] and are most likely due to IGF1 deficiency and the lack of its protein anabolic action (reviewed in [73]).

Since the liver is a major target organ for GH, we investigated alterations in the activity of selected hepatic signaling pathways in adult fasted *GHR*-KO and control pigs. Upon binding of GH, the GHR homodimer undergoes a conformational change that brings the originally parallel receptor transmembrane domains into a rotated crossover orientation, thus separating the lower parts of the transmembrane helices. Thereby, the two JAK2 molecules that are associated with the membrane proximal Box1 motif of the GHR chains are separated, and the inhibitory pseudokinase domain of one JAK2 is removed from the kinase domain of the other JAK2 and vice versa [74]. The kinase domains of the two JAK2 molecules can then be transactivated and initiate tyrosine phosphorylation of the GHR cytoplasmic domains and STAT5, the key transcription factor mediating most genomic actions of GH (reviewed in [75]), including stimulation of *IGF1* gene expression [76]. In agreement with this concept, the phosphorylation of STAT5 in liver of *GHR*-KO pigs was significantly reduced and the circulating IGF1 levels were markedly decreased compared with control pigs. However, unexpectedly, phosphorylation of JAK2 was significantly increased in liver of *GHR*-KO pigs, together with a significant increase in the phosphorylation of MAPK, PI3K, and mTOR, which are known to be – directly or indirectly – activated by JAK2 [77].

In addition to GHR, many other class I cytokine receptors also use the non-receptor tyrosine kinase JAK2 for signaling, including the receptors for erythropoietin, prolactin, interleukins 3, 5 and 6, granulocyte-macrophage colony-stimulating factor, interferon-gamma, thrombopoietin, and leptin (reviewed in [78]). GHR is abundantly expressed in liver and can dimerize and associate with JAK2 in the absence of GH (reviewed in [75]). Since GHR-bound JAK2 can only be activated in the presence of GH, elimination of GHR may increase the pool of JAK2 that can be phosphorylated by other class I cytokine receptors. In addition, the loss of GHR may alter the abundance of these receptors or their ligands and thus lead to increased JAK2 phosphorylation.

A candidate is leptin, since increased serum leptin levels have been detected in LS patients [79] and in the *Ghr* KO mouse model (reviewed in [16]). Leptin is known to induce the expression of its receptor in liver [80], and leptin receptor activation involves the phosphorylation of JAK2 and STAT3, but not STAT5 (reviewed in [37]). Consistent with this hypothesis, we observed significantly increased serum leptin levels and increased expression and phosphorylation of leptin receptors in liver of *GHR*-KO pigs, providing a potential explanation for the significantly increased phosphorylation of JAK2, while phosphorylation of STAT5 was significantly reduced.

Phosphorylation of the serine/threonine kinase mTOR that forms the catalytic core of mTOR complex-1 (mTORC1) and mTORC2 was significantly increased in *GHR*-KO pigs. While mTOR phosphorylation was not directly investigated in *Ghr* KO mice, Dominick et al. [35] have evaluated the activities of mTORC1 and mTORC2 in fed and fasted animals based on the phosphorylation status of downstream substrates of these complexes. In fasted *Ghr* KO mice, the authors observed reduced phosphorylation of the ribosomal protein S6, while the phosphorylation status of S6K and 4EBP1 was unaltered compared with control mice. In fed *Ghr* KO mice, the phosphorylation status of all three indicators of mTORC1 activity was significantly reduced. In contrast, the phosphorylation level of several target substrates of mTORC2, including AKT, was significantly increased in fasted *Ghr* KO vs. control mice, while this difference disappeared after feeding [35]. The authors concluded that mTORC2 activity was increased in *Ghr* KO mice – at least in fasted animals — whereas mTORC1 activity was unaltered or reduced in fasted and fed *Ghr* KO mice, respectively. Our study of liver samples from fasted *GHR*-KO pigs did not reveal an increase in the phosphorylation of S6K or of 4EBP1 that would trigger its dissociation from eIF4E and allow cap-dependent mRNA translation (reviewed in [36]). Moreover, we observed a significant increase in phosphorylation (= activation) of the mTORC1 inhibitor AMPK [81]. Collectively, these findings argue against a major activation of mTORC1 in liver of *GHR*-KO pigs. In contrast, a significant increase in GSK3B phosphorylation and a trend (p = 0.0635) toward an increase in AKT phosphorylation suggested a rise in the activity of mTORC2. While mTORC1 has profound effects on mRNA translation, metabolism, and protein turnover, mTORC2 signaling is implicated in the regulation of ion transport, apoptosis, glucose metabolism, cell migration, and cytoskeleton rearrangement (reviewed in [36]). Future molecular profiling studies of liver and other tissues from *GHR*-KO and control pigs will clarify whether these biological processes are altered in the absence of GHR signaling.

Another interesting observation in liver samples from *GHR*-KO pigs was the trend (p = 0.0635) toward an increased abundance of PPARG. Discrepant findings have been reported regarding the consequences of increased PPARG activity in liver, ranging from the promotion of hepatic steatosis through the upregulation of genes involved in lipid uptake and storage, to the prevention of hepatic steatosis and fibrosis, possibly by sequestering fatty acids in adipose tissue and preventing hepatic stellate cell activation (reviewed in [82]). Studies of *GHR*-KO and control pigs, e.g., after being fed a high-fat diet, may provide additional insight into these mechanisms.

One of the most striking effects of GHR deficiency is the increased life-span that has been observed in *Ghr* KO mice [83], [84]. Moreover, patients with LS show reduced incidences of cancer and diabetes, associated with reduced pro-aging signaling in cells incubated with serum derived from these patients [7]. While life expectancy studies in humans are difficult due to their long duration and to multiple confounding factors, they are possible in pigs, which can be maintained under standardized conditions and have a normal life expectancy of 15–17 years (reviewed in [85]). Moreover, protective effects of GHR deficiency against tumors and diabetes can be evaluated by crossing the *GHR* KO mutation in existing pig models that are genetically predisposed to tumor development (e.g., [86]) or (pre-)diabetes [27], [87], thus bridging the gap between rodents and humans in longevity research with tailored large animal models. In addition to these long-lasting *in vivo* experiments, the effects of GHR deficiency on resistance to stress – such as UV light and heat – may be evaluated in primary cell cultures from *GHR*-KO pigs, similarly to studies that have been performed using cultured cells from dwarf mice [88].

Investigation of the neurological consequences of GHR deficiency is another interesting field, since mental retardation has been reported in a proportion of LS patients [2], while subjects from the Ecuadorian cohort display normal intelligence [89] or even enhanced cognitive performance [90]. As sophisticated methods for testing cognitive functions of pigs are available [91], the *GHR*-KO pig may also serve as a model to address these questions.

Finally, *GHR*-KO pigs are interesting animal models for developing and evaluating the efficacy and safety of new treatment options for LS, such as PASylated IGF1 with a prolonged plasma half-life [92]. In addition, it would be interesting to determine whether the phenotype of *GHR*-KO pigs can be rescued by correction of the *GHR* mutation in a proportion of liver cells via gene editing. In a mouse model, hydrodynamic injection into the tail vein has been used to deliver components of a CRISPR/Cas9 system to correct a mutated fumarylacetoacetate hydrolase (*Fah*) gene in hepatocytes *in vivo* [93]. In pigs, refined techniques are available for direct application into the liver [94], [95].

In summary, G*HR*-KO pigs resemble important aspects of the pathophysiology of human Laron syndrome and are thus an interesting model for mechanistic studies and treatment trials.

## Funding

This study was supported in part by the German Center for Diabetes Research (DZD) and by the German Research Council (TRR127).

## Author contributions

A. Hi., B. K., M. D., and E. W. conceived the experiments. A. Hi. and E. W. wrote the manuscript. All authors contributed to the manuscript and read and approved the final version. M. D., S. B., and H. L. developed the CRISPR/Cas system for *GHR* KO. B. K., M. K., and H. N. performed the *in vitro* fertilization, microinjection and embryo transfer experiments; A. Hi. and B. K. managed the breeding and performed the phenotypic characterization of *GHR*-KO and control pigs. A. Hi., A. B., and R. W. performed the necropsies. E. K. conducted the GHR ligand immunohistochemistry. M. B. and A. M. S. performed the DXA and MRI studies. A. Ho. carried out the IGFBP ligand blots analyses. W. F. B. and M. B. performed the IGF1 and GH assays. S. R. performed the insulin and glucose measurements, B. R. and M. H. d. A. the clinical-chemical measurements. M. D. performed the Western blot analysis of signaling molecules. A. Hi. and E. W. are the guarantors of this work and, as such, had full access to all the data in the study and take responsibility for the integrity of the data and the accuracy of the data analysis.

## Appendix A Supplementary data

mmc1
