# Supplementary material for: Growth hormone receptor-deficient pigs resemble the pathophysiology of human Laron syndrome and reveal altered activation of signaling cascades in the liver
Source: Mol Metab. 2018 Mar 15;11:113–28. doi: 10.1016/j.molmet.2018.03.006 (PMC6001387; doi:10.1016/j.molmet.2018.03.006)
Supplement: mmc1 [file mmc1.pdf]

Supplementary Material

Growth hormone receptor-deficient pigs resemble the pathophysiology of human Laron syndrome and reveal altered activation of signaling cascades in the liver

Arne Hinrichs, Barbara Keßler, Mayuko Kurome, Andreas Blutke, Elisabeth Kemter, Maren Bernau, Armin M. Scholz, Birgit Rathkolb, Simone Renner, Sebastian Bultmann, Heinrich Leonhardt, Martin Hrabě de Angelis, Hiroshi Nagashima, Andreas Hoeflich, Werner F. Blum, Martin Bidlingmaier, Rüdiger Wanke, Maik Dahlhoff, Eckhard Wolf

Supplementary Table 1. Antibodies employed for Western blot analyses.

Antigen	Antibody	Host	Dilution
pJAK2	Cell Signaling, Frankfurt, Germany #3776	rabbit	1:2000
JAK2	Cell Signaling, Frankfurt, Germany #3230	rabbit	1:2000
pMAPK	Cell Signaling, Frankfurt, Germany #4370	rabbit	1:2000
MAPK	Cell Signaling, Frankfurt, Germany #9102	rabbit	1:2000
pSTAT1	Cell Signaling, Frankfurt, Germany #7649	rabbit	1:2000
STAT1	Cell Signaling, Frankfurt, Germany #14994	rabbit	1:2000
pSTAT3	Cell Signaling, Frankfurt, Germany #9145	rabbit	1:2000
STAT3	Cell Signaling, Frankfurt, Germany #12640	rabbit	1:2000
pSTAT5	Cell Signaling, Frankfurt, Germany #9359	rabbit	1:2000
STAT5	Cell Signaling, Frankfurt, Germany #94205	rabbit	1:2000
pPI3K	Cell Signaling, Frankfurt, Germany #4228	rabbit	1:2000
PI3K	Cell Signaling, Frankfurt, Germany #4257	rabbit	1:2000
pAKT	Cell Signaling, Frankfurt, Germany #4060	rabbit	1:2000
AKT	Cell Signaling, Frankfurt, Germany #4691	rabbit	1:2000
pmTOR	Cell Signaling, Frankfurt, Germany #5536	rabbit	1:1000
mTOR	Cell Signaling, Frankfurt, Germany #2983	rabbit	1:1000
pAMPK	Cell Signaling, Frankfurt, Germany #2535	rabbit	1:2000
AMPK	Cell Signaling, Frankfurt, Germany #2532	rabbit	1:2000
pS6K	Cell Signaling, Frankfurt, Germany #9205	rabbit	1:2000
pS6K	Cell Signaling, Frankfurt, Germany #2708	rabbit	1:2000
p4EBP1	Cell Signaling, Frankfurt, Germany #2855	rabbit	1:2000
4EBP1	Cell Signaling, Frankfurt, Germany #9644	rabbit	1:2000
eIF4E	Cell Signaling, Frankfurt, Germany #9742	rabbit	1:2000
PPARG	Cell Signaling, Frankfurt, Germany #2435	rabbit	1:2000
pGSK3B	Cell Signaling, Frankfurt, Germany #9322	rabbit	1:2000
GSK3B	Cell Signaling, Frankfurt, Germany #9315	rabbit	1:2000
pINSR	Cell Signaling, Frankfurt, Germany #3024	rabbit	1:2000
INSR	Cell Signaling, Frankfurt, Germany #3025	rabbit	1:2000
pIRS1	Cell Signaling, Frankfurt, Germany #3203	rabbit	1:2000
IRS1	Cell Signaling, Frankfurt, Germany #3407	rabbit	1:2000
pLEPR	Merck, Darmstadt, Germany #07-1317	rabbit	1:500
LEPR	Bio-Rad, Munich, Germany #AHP1396	goat	1:1000

Supplementary Table 2. Factors affecting body fat content, muscle-to-back fat ratio, and serum leptin levels of 6-month-old control and *GHR*-KO pigs.

Parameter	Control			*GHR*-KO			Factors (p values)		
	Sex	mean	SD	Sex	mean	SD	Group	Sex	Group*Sex
Body fat (%)	male	9.6	1.1	male	20.9	2.2	< 0.0001	0.0108	0.1543
	female	13.2	2.5	female	22.0	4.1			
Muscle-to-back fat ratio (v/v)	male	8.6	2.3	male	2.9	0.5	< 0.0001	0.6289	0.4335
	female	7.7	2.3	female	3.1	1.3			
Serum leptin (µg/L)	male	3.0	1.0	male	4.8	2.7	0.0066	0.013	0.7437
	female	4.7	1.2	female	6.1	1.6			

The table shows means and standard deviations (SD) of means for body fat (%) and muscle to fat ratio calculated for 11 male/14 female control pigs and for 6 male/6 female *GHR*-KO pigs. Means and SD for fasting serum leptin were calculated for 9 male/13 female control and 6 male/6 female *GHR*-KO pigs. Data were analyzed using the General Linear Models Procedure (PROC GLM) as described in section 2.8 of the main manuscript.

Supplementary Table 3. Organ weights of control and *GHR*-KO pigs.

	Control (n = 25)	*GHR*-KO (n = 9)	% of Control	p value
Absolute weights (g)				
Liver	2315.5 ± 80.4	711.5 ± 133.9	30.6 ± 1.8	< 0.0001
Kidneys*	335.7 ± 11.0	102.4 ± 18.4	30.7 ± 1.4	< 0.0001
Heart	356.6 ± 9.9	133.0 ± 16.4	37.3 ± 2.1	< 0.0001
Brain	99.3 ± 1.5	83.4 ± 2.5	84.1 ± 2.0	< 0.0001
Lungs	686.2 ± 21.6	257.6 ± 34.9	37.1 ± 2.2	< 0.0001
Spleen	491.6 ± 28.9	177.6 ± 48.0	36.1 ± 4.1	< 0.0001
Pituitary gland	0.27 ± 0.01	0.13 ± 0.01	46.2 ± 1.8	< 0.0001
Adrenal glands*	5.56 ± 0.30	2.10 ± 0.50	37.0 ± 3.0	< 0.0001
Thyroid gland	7.45 ± 0.30	3.05 ± 0.60	40.4 ± 2.6	< 0.0001
Relative weights (% of body weight)				
Liver	2.53 ± 0.10	1.85 ± 0.20	72.8 ± 2.8	0.0005
Kidneys*	0.37 ± 0.01	0.27 ± 0.02	74.1 ± 3.8	0.0002
Heart	0.39 ± 0.01	0.34 ± 0.01	89.1 ± 2.1	0.0119
Brain	0.11 ± 0.005	0.22 ± 0.01	200.7 ± 10.0	< 0.0001
Lungs	0.75 ± 0.03	0.68 ± 0.05	89.3 ± 6.7	0.2304
Spleen	0.54 ± 0.03	0.46 ± 0.06	86.1 ± 9.2	0.2525
Pituitary gland	2.90 E-4 ± 0.09 E-4	3.30 E-4 ± 0.15 E-4	110.2 ± 4.0	0.0899
Adrenal glands*	6.10 E-3 ± 0.36 E-3	5.50 E-3 ± 0.65 E-3	88.7 ± 5.6	0.4267
Thyroid gland	8.20 E-3 ± 0.45 E-3	8.20 E-3 ± 0.80 E-3	98.2 ± 7.7	0.9384

Data are presented as least squares means (LSM) and standard errors (SE) of LSM estimated for the 2 groups (the statistical model used is described in section 2.8 of the main manuscript). LSMs were compared using Student's *t*-tests. *Cumulative weight of both organs.

Supplementary Table 4. Factors affecting parameters of lipid metabolism of control and *GHR*-KO pigs.

Parameter	Age	Sex	Control		*GHR*-KO		Factors (p values)				
			mean	SD	mean	SD	Group	Sex	Age	Group*Age	Group*Sex*Age
Triglycerides (mg/dL)	1	male	40.3	16.5	20.5	6.1	0.0113	0.2435	0.7666	0.301	0.9918
		female	44.3	20.5	25.8	10.9					
	2	male	34.8	14.6	26.2	8.1					
		female	42.1	20.3	33.9	11.0					
Cholesterol (mg/dL)	1	male	76.6	7.9	61.6	4.6	0.0851	< 0.0001	0.0535	0.0291	0.6255
		female	86.9	4.7	78.4	4.8					
	2	male	60.4	11.4	60.6	7.9					
		female	78.2	10.4	81.0	6.7					
LDL (mg/dL)	1	male	47.1	5.8	38.3	3.9	0.0971	< 0.0001	0.1097	0.1181	0.6526
		female	53.2	3.1	47.4	2.9					
	2	male	36.7	8.1	36.6	5.1					
		female	49.2	9.8	48.9	3.4					
HDL (mg/dL)	1	male	31.2	3.0	26.6	4.6	0.7111	0.0002	0.5071	0.0022	0.4577
		female	36.5	3.2	31.4	4.2					
	2	male	26.9	5.1	28.1	4.7					
		female	30.3	2.2	36.9	2.9					

The table shows means and standard deviations (SD) of means calculated for 6 male/4 female control pigs and 3 male/3 female *GHR*-KO pigs at Age 1 (12-15 weeks) and for 8 male/6 female control pigs and 5 male/4 female *GHR*-KO pigs at Age 2 (23-27 weeks). Data were analyzed using the General Linear Models Procedure (PROC GLM) as described in section 2.8 of the main manuscript.

Supplementary Table 5. Clinical-chemical parameters of 6-month-old control and *GHR*-KO pigs.

Parameter [unit]	Control (n = 14)	*GHR*-KO (n = 9)	p value	Normal range
Urea [mmol/L]	4.3 ± 0.3	7.4 ± 0.5	< 0.0001	3.2 – 8.2
Creatinine [µmol/L]	126.4 ± 5.0	91.2 ± 6.5	0.0004	40 – 133
Total protein [g/L]	65.3 ± 2.2	63.6 ± 2.9	0.6381	55 – 85
Albumin [g/L]	40.1 ± 1.4	39.8 ± 1.8	0.8957	17.3 – 43.3
Bilirubin [µmol/L]	1.8 ± 0.1	1.5 ± 0.2	0.2351	< 4.3
GGT [U/L]	36.4 ± 1.8	32.5 ± 2.4	0.2131	0 – 40
Sodium [mmol/L]	141.5 ± 1.0	139.9 ± 1.3	0.3330	133 – 150
Potassium [mmol/L]	5.0 ± 0.2	4.2 ± 0.3	0.0602	4.4 – 6.7
Chloride [mmol/L]	101.8 ± 0.9	99.6 ± 1.2	0.1586	95 – 110
Calcium [mmol/L]	2.6 ± 0.04	2.7 ± 0.05	0.1744	2.4 – 3.0
Magnesium [mmol/L]	1.1 ± 0.04	1.1 ± 0.05	0.2740	0.5 – 1.2
Phosphorus [mmol/L]	2.4 ± 0.1	2.2 ± 0.04	0.1063	2.1 – 3.3
Iron [µmol/L]	17.7 ± 1.2	23.6 ± 1.6	0.0094	> 17.9

Data are presented as least squares means (LSM) and standard errors (SE) of LSM estimated for the 2 groups. The statistical model used is described in section 2.8 of the main manuscript. LSM were compared using Student's t-test.

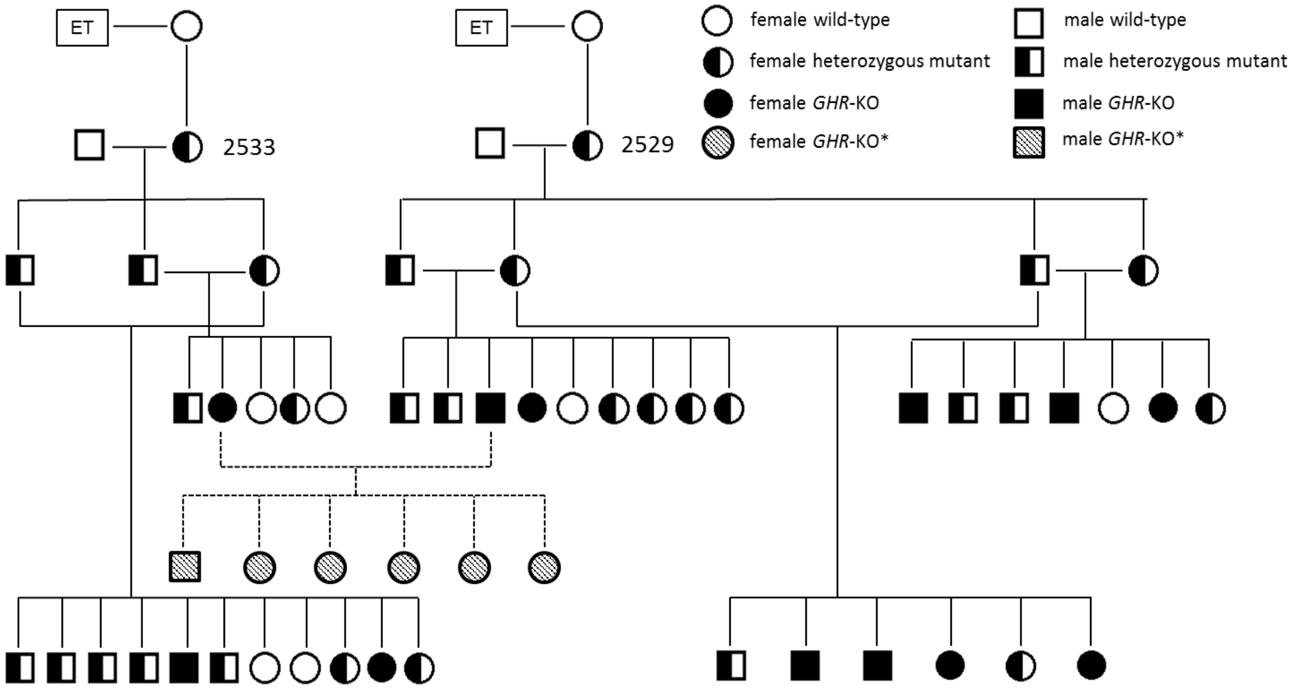

Supplementary Figure 1. Breeding scheme of *GHR*-KO animals. Heterozygous founder animals 2533 and 2539 were mated with wild-type boars and their heterozygous offspring were intercrossed to produce *GHR*-KO animals as well as wild-type and heterozygous *GHR* mutant littermates. The mating of two *GHR*-KO animals resulted in a litter of six healthy *GHR*-KO* piglets.

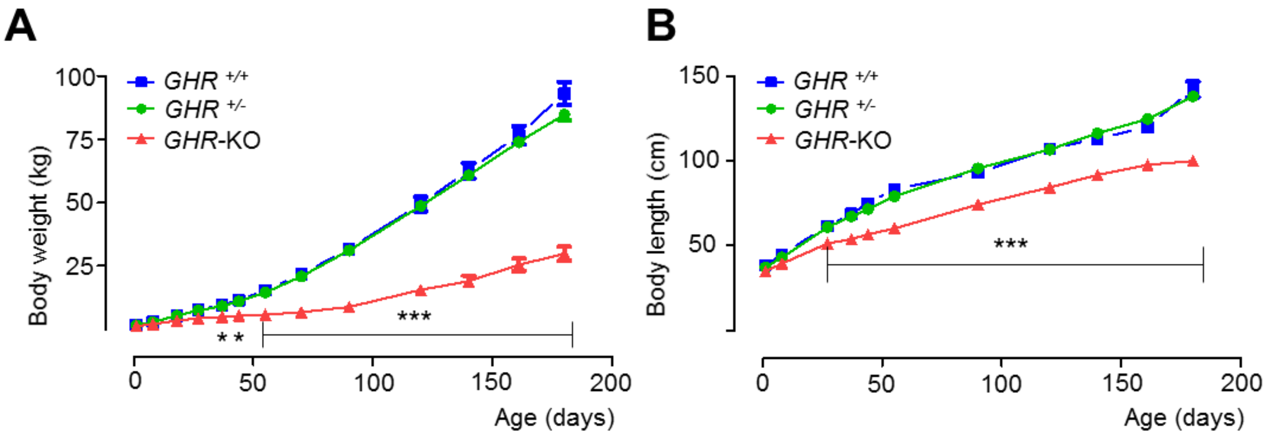

Supplementary Figure 2. Body weight gain (A) and longitudinal growth (B) of *GHR*-KO (n = 12) compared to wild-type (*GHR* $^{+/+}$; n = 6) and heterozygous *GHR* mutant (*GHR* $^{+/-}$; n = 19) pigs. Note that there was no significant difference in growth of *GHR* $^{+/+}$ and *GHR* $^{+/-}$ pigs. The two groups were thus pooled as GHR expressing control group.

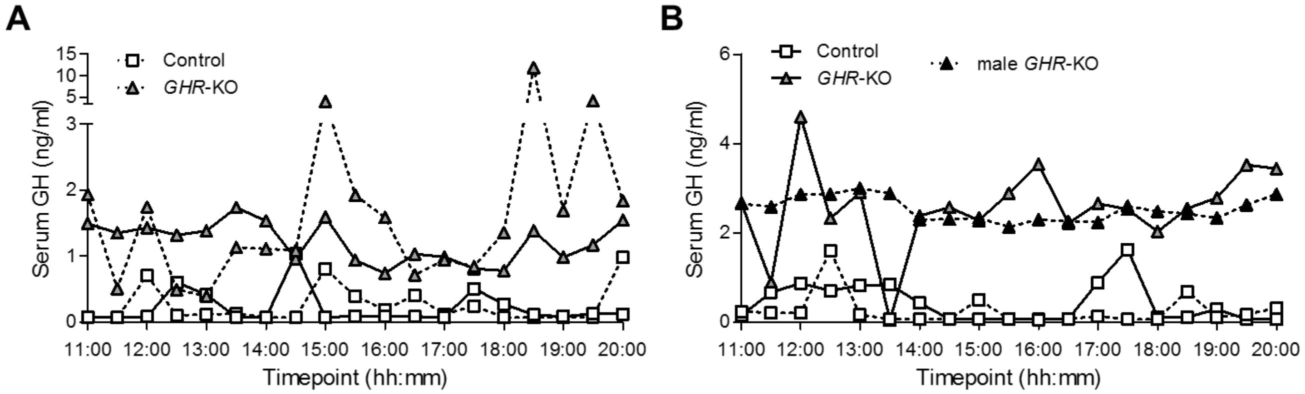

Supplementary Figure 3. GH secretion profiles of additional *GHR*-KO (n=4) and control pigs (n=4). (A) Secretion pattern of two female *GHR*-KO and control pigs. (B) Secretion pattern of a female and male *GHR*-KO and two female control pigs.

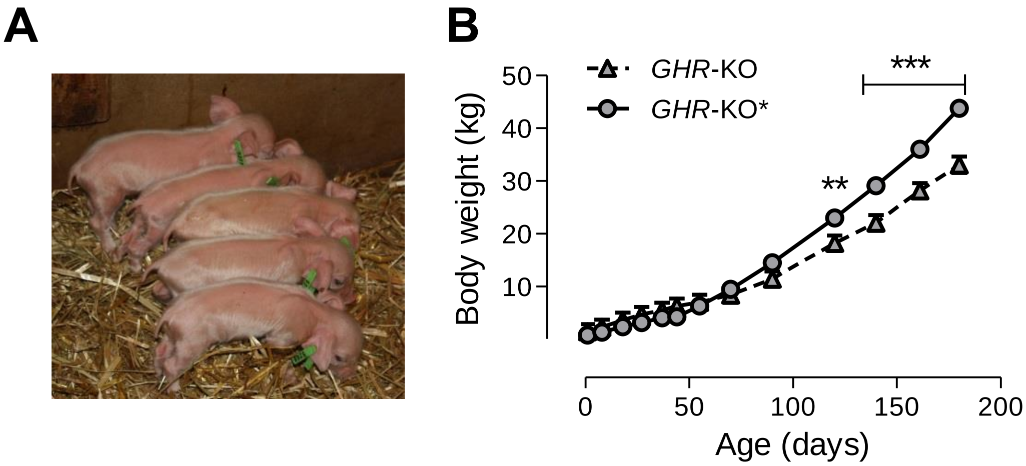

Supplementary Figure 4. *GHR*-KO animals are able to reproduce. Mating of a *GHR*-KO sow and *GHR*-KO boar resulted in a litter of healthy *GHR*-KO* piglets. **(A)** Picture of *GHR*-KO* piglets at the age of one day. **(B)** *GHR*-KO* piglets had lower birth weights than *GHR*-KO piglets from heterozygote x heterozygote mating, but reached a higher weight at 6 months of age (*GHR*-KO: n = 12, *GHR*-KO*: n = 6). Data are presented as least squares means (LSM) and standard errors (SE) of LSM estimated for the 2 groups. The statistical model used is described in section 2.8 of the main manuscript. LSMs were compared using Student's t-test.

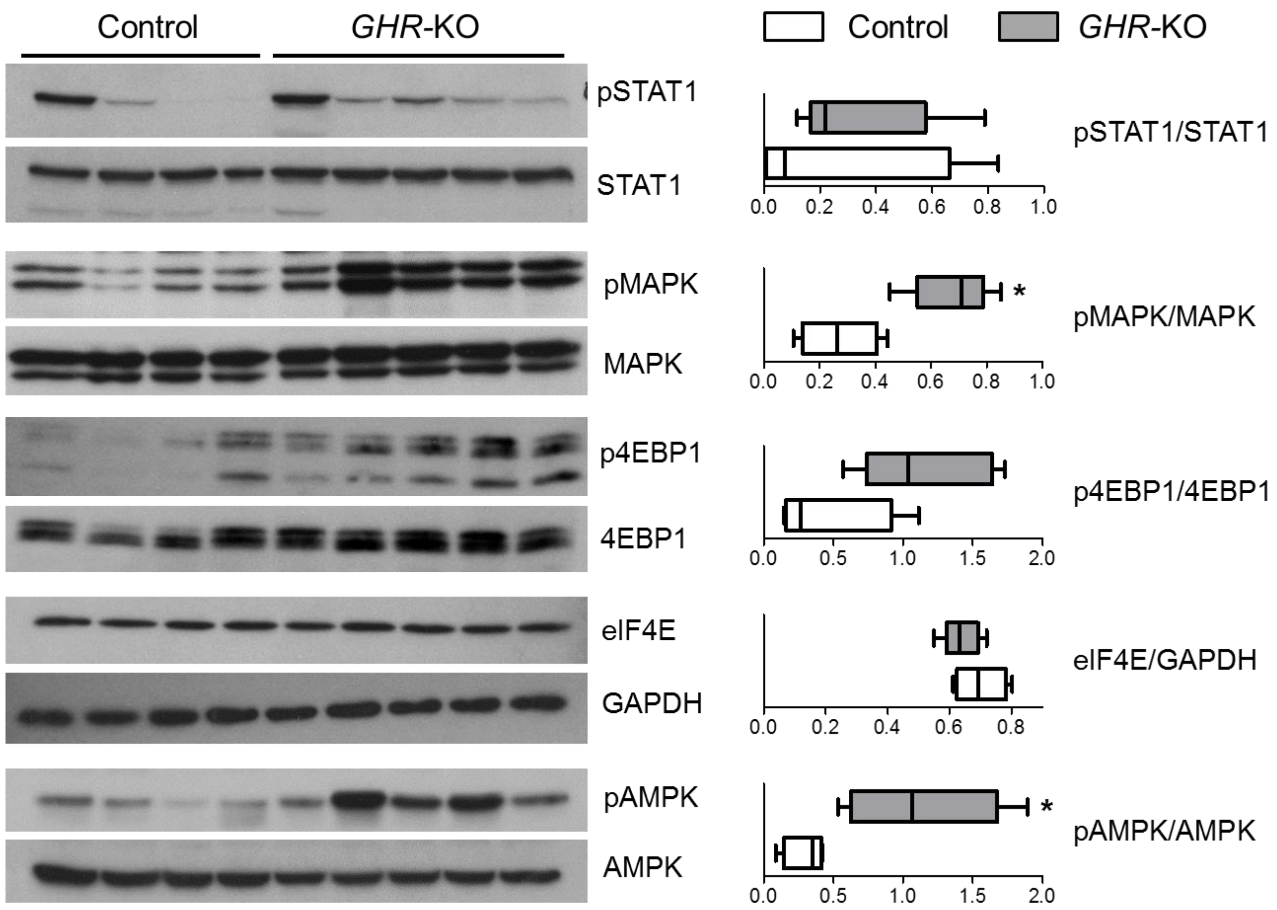

Supplementary Figure 5. Western blot analysis of GHR-related signaling molecules in liver samples of 6-month-old fasted *GHR*-KO (n=5) and control pigs (n=4). The box plots show medians, 25th and 75th percentiles (box), and extremes (whiskers). * $p < 0.05$; evaluated using Mann-Whitney U test.
